# Reduced peroxisomal function increases insulin secretion, promotes insulin oxidation, and impairs **β** cell maturity

**DOI:** 10.1172/jci.insight.200202

**Published:** 2026-06-30

**Authors:** J. Jason Collier, Caroline R. Cothern, Maggie P. Ducote, Thomas M. Martin, Melissa A. Linden, Robert C. Noland, David H. Burk, Samuel D. Dupuy, Michael D. Karlstad, Krisztian Stadler, Sarah S. Hirschbeck, Thanh D. Do, Anastasia Coldren, Marcela Brissova, Teayoun Kim, Kirk M. Habegger, Sujoy Ghosh, Zane A. Vickery, Qudus Sarumi, Shawn R. Campagna, Susan J. Burke

**Affiliations:** 1 Pennington Biomedical Research Center, Baton Rouge, Louisiana, USA.; 2 Department of Biological Sciences, Louisiana State University, Baton Rouge, Louisiana, USA.; 3 Department of Surgery, The University of Tennessee Graduate School of Medicine, Knoxville, Tennessee, USA.; 4 Department of Chemistry, The University of Tennessee Knoxville, Tennessee, USA.; 5 Department of Medicine, Division of Diabetes, Endocrinology, and Metabolism, Vanderbilt University Medical Center, Nashville, Tennessee, USA.; 6 Comprehensive Diabetes Center, The University of Alabama at Birmingham, Birmingham, Alabama, USA.

**Keywords:** Endocrinology, Metabolism, Fatty acid oxidation, Insulin, Islet cells

## Abstract

Given the central role of peroxisomes in lipid metabolism and redox homeostasis, we hypothesized that peroxisomal activity is critical for sustaining β cell function and identity. Pex5 deletion models were employed to investigate the loss of peroxisomal function on glucose-stimulated insulin secretion (GSIS), oxidative stress, and β cell maturity markers. Peroxisome deficiency in male mice resulted in elevated GSIS. Glucose intolerance developed despite increased insulin secretion. Ion mobility mass spectrometry revealed oxidation of insulin proteins and a truncated insulin 2–derived peptide in islets from mice with a tissue-specific deficiency in peroxisomes. Peroxisome loss of function increased multiple markers of oxidative stress, including altered metabolite profiles, lipid peroxidation, and protein carbonylation. These findings revealed that increased secretion of oxidized insulin protein is insufficient to regulate whole-body glucose homeostasis. Peroxisome deficiency also reduced markers of β cell maturity. Based on these outcomes, we identified the peroxisome organelle as a key regulatory component of glucose homeostasis by protecting insulin from oxidative modification and degradation and by supporting maintenance of mature β cells.

## Abstract 2

<span>Small cellular structures called peroxisomes are essential for insulin-producing cells to work properly. Without them, insulin release and cell maturity become impaired.</span>

## Introduction

Peroxisomes, first identified as microbodies over 70 years ago, are dynamic organelles that play essential roles in cellular lipid metabolism and redox homeostasis (1, 2). They mediate β-oxidation of very-long-chain fatty acids, the metabolism of branched-chain and ether-linked lipids, and detoxification of hydrogen peroxide through antioxidant enzymes. Beyond these canonical functions, peroxisomes are increasingly recognized as hubs of cellular communication, influencing mitochondrial metabolism, redox signaling, and innate immunity (3). Human peroxisomal biogenesis disorders highlight the importance of these organelles, as defects in matrix protein import or enzymatic activity cause severe systemic metabolic dysfunction. Moreover, altered peroxisomal metabolism is implicated in common diseases, including neurodegeneration, obesity, and diabetes (2).

Peroxisome contribution to pancreatic β cell physiology, where they are abundantly expressed (4, 5), remains incompletely understood. β cells maintain whole-body glucose homeostasis by secreting insulin in response to nutrient stimulation, a process dependent on both glucose metabolism and lipid-derived signals (6, 7). Acute increases in fatty acid availability potentiate GSIS, whereas chronic ectopic lipid accumulation in pancreatic tissue disrupts β cell function and contributes to type 2 diabetes in humans and rodents (8–10). β cells are highly vulnerable to oxidative stress, which damages cellular macromolecules and compromises their health and function (11). Given the dual roles in lipid metabolism and ROS detoxification, peroxisomes are uniquely positioned to protect β cells against metabolic and oxidative stress. However, direct evidence linking peroxisomal function to β cell physiology remains limited (4).

Peroxins are proteins involved in peroxisomal biogenesis, among which Pex5 functions as the cytosolic receptor for peroxisomal targeting signal 1–containing proteins and is essential for the import of most matrix enzymes (12). Loss of Pex5 function results in structurally intact but enzymatically inactive organelles, effectively modeling peroxisome deficiency (13). Here, we describe mouse models generated specifically to investigate the role of peroxisomes to support β cell function and maturity using both pancreas- and β cell–specific Pex5 gene deletion. We found that peroxisomal deficiency leads to elevated GSIS but paradoxically impaired glucose tolerance. Our results uncover a role for peroxisomes to maintain integrity of the insulin protein, sustain markers of mature β cells, and prevent pathophysiological oxidative stress within islet β cells.

## Results

### Pancreas- and β cell–specific deletion of Pex5 impairs peroxisomal matrix protein import and peroxisome function.

Peroxisomes are abundant in β cells (4,5), but their role in β cell function and maturity is unclear. Because Pex5 is required for importing the majority of peroxisomal proteins into the peroxisomal matrix, its deletion disrupts peroxisomal function (13). Therefore, using both the Pdx1-Cre and Ins1-Cre driver lines, we generated mice with a pancreas-specific (Figure 1, A–C) or β cell–specific deficiency (Figure 1, I–K) in Pex5 expression and abundance. The control group (Pex5^CON^) comprised littermates with the following genotypes: (a) non-floxed, non-Cre–expressing mice; (b) Pex5 floxed, non-Cre–expressing mice; and (c) Cre-expressing, non-floxed mice. These groups were combined in our analyses to increase rigor because no differences were observed among control genotypes for any parameter examined (glucose tolerance test shown in Supplemental Figure 1, A and B, as an example; supplemental material available online with this article; https://doi.org/10.1172/jci.insight.200202DS1).

Immunostaining for the peroxisomal membrane protein PMP70 revealed that PMP70-positive peroxisomal structures were markedly more abundant in islet cells than acinar cells of Pex5^CON^ and Pex5^Pdx1–/–^ mice, indicating that β cells are the major cell type affected by Pdx1-Cre–mediated recombination (Figure 1D). However, the presence of PMP70-positive structures does not differentiate between import-competent, functional peroxisomes and import-deficient residual structures, referred to as peroxisome ghosts, a known feature of the Pex5 deletion model (13). In β cells from Pex5^CON^ mice, colocalization of PMP70 with the matrix protein, Acox1, indicated the presence of import-competent, functional peroxisomes, identified by yellow puncta (Figure 1E). In contrast, Acox1 and PMP70 colocalization was markedly reduced in β cells from Pex5^Pdx1–/–^ mice (Figure 1E), consistent with a substantial reduction in the formation of import-competent, functional peroxisomes.

Impaired peroxisomal matrix protein import and reduced biogenesis are expected to disrupt lipid metabolism pathways, leading to alterations in lipid profiles. Consistent with this, we observed a broad reduction in the concentration of multiple ether phospholipid and ether lysophospholipid species in male Pex5^Pdx1–/–^ mice compared with controls (Figure 1F). Loss of peroxisomal function, and more specifically altered lipid profiles, did not affect serum triglyceride levels (Figure 1G) or peripheral insulin sensitivity assessed by an insulin tolerance test (ITT; Figure 1H) in male Pex5^Pdx1–/–^ mice. Similarly, in male Pex5^Ins1–/–^ mice (Figure 1, I–M), the yellow arrow indicates PMP70/Acox1 colocalization in Pex5^CON^ mice and the red arrow indicates a β cell in Pex5^Ins1–/–^ mice with vastly reduced colocalization (Figure 1K), but serum triglyceride levels (Figure 1L) and insulin sensitivity (Figure 1M) were comparable between genotypes.

Body mass and composition were assessed in male mice from 8 to 30 weeks and female mice from 12 to 36 weeks. Male Pex5^Pdx1–/–^ mice exhibited increased body mass (Supplemental Figure 2A), fat mass (Supplemental Figure 2B), and fluid mass (Supplemental Figure 2C) by 10 weeks of age, with no change in lean mass (Supplemental Figure 2D). In a separate cohort of male mice, we evaluated whether the increased adiposity seen in Pex5^Pdx1–/–^ mice could be linked to changes in energy expenditure, respiratory quotient, caloric intake, or activity levels (Supplemental Figure 2, E and F). These measurements took place at 22 weeks of age when differences in body mass and fat mass were already established. Increased energy expenditure in Pex5^Pdx1–/–^ mice was most likely due to the increase in body mass, as there were no discernible differences in food or liquid intake (Supplemental Figure 2, G and H), mean respiratory quotient (Supplemental Figure 2, I and J), total activity (Supplemental Figure 2K), or mean sleep time (Supplemental Figure 2L) between Pex5^CON^ and Pex5^Pdx1–/–^ mice.

Similarly, female Pex5^Pdx1–/–^ mice exhibited increased adiposity compared with Pex5^CON^ (Supplemental Figure 3, A–D). In contrast, no differences in body composition were observed across the lifespan of Pex5^Ins1–/–^ male (Supplemental Figure 2, A–D) or female (Supplemental Figure 3, A–D) mice compared with Pex5^CON^. Similar to male mice, female Pex5^Pdx1–/–^ and Pex5^Ins1–/–^ mice showed no alteration in peripheral insulin sensitivity (Supplemental Figure 3E) or serum triglyceride concentration (Supplemental Figure 3F).

### Peroxisomes are critical for maintenance of glucose tolerance and β cell function in male mice.

Pex5 heterozygous KO mice (Pex5^Pdx1+/–^) displayed normal glucose tolerance compared with controls (Supplemental Figure 1, B and C). Although postprandial blood glucose was unchanged (Figure 2A), male Pex5^Pdx1–/–^ mice exhibited impaired glucose tolerance compared with Pex5^CON^ mice (Figure 2, B–F). Glucose tolerance tests (GTTs) i.p. administered to male Pex5^Pdx1–/–^ mice at 8, 12, 20, and 30 weeks of age (Figure 2, B–E), demonstrated that glucose intolerance manifests early in life, even prior to alterations in body composition (Supplemental Figure 2, A–D). The phenotype was also consistent in male Pex5^Pdx1–/–^ mice during an oral GTT (Figure 2F).

We next examined GSIS both in vivo by hyperglycemic clamp and ex vivo by perifusion using isolated islets. During the clamp, blood glucose was maintained at approximately 250 mg/dL for 120 minutes and did not differ between the genotypes (Figure 2G). No difference was observed in the glucose infusion rate (Figure 2H). Surprisingly, plasma insulin levels were significantly higher in Pex5^Pdx1–/–^ mice compared with controls in response to hyperglycemia (Figure 2I), indicating greater insulin output in vivo. Significant increases in both glucose- and KCl-stimulated insulin secretion were observed in 12-week-old (Figure 2, J and K) and 30-week-old (Figure 2, L and M) male Pex5^Pdx1–/–^ mice compared with controls. Despite elevations in insulin secretion, insulin content remained unchanged (Figure 2N). Glucose intolerance by GTT administered i.p. (Figure 2O) and oral GTT (Figure 2P) was also observed in male Pex5^Ins1–/–^ mice, with a similar increase as Pex5^Pdx1–/–^ mice in both glucose-stimulated and KCl-mediated insulin secretion compared with controls (Figure 2, Q–S). Consistent with the ex vivo data from mouse islets (2.5-, 2.8-, and 3-fold in Figure 2, K, M, and R, respectively), GSIS was elevated by 1.9-fold in Pex5-deficient 832/13 insulinoma cells (Figure 2T). Collectively, these data demonstrate that impaired peroxisome function increases β cell insulin secretion but leads to impaired glucose tolerance.

### Reduced peroxisomal function in the β cells of female mice has no impact on β cell function.

Postprandial blood glucose values were comparable across all female groups until 30 weeks of age, at which point Pex5^Pdx1–/–^ mice exhibited a modest but significant increase compared with controls (Figure 3A). Both Pex5^Pdx1–/–^ and Pex5^Ins1–/–^ females remained glucose-tolerant early in life, as evidenced from GTTs i.p. administered at 12 and 20 weeks of age (Figure 3, B and C); however, Pex5^Pdx1–/–^ mice displayed an age-dependent decline in glucose tolerance compared with controls by 34 weeks of age (Figure 3D). This phenotype was confirmed in Pex5^Pdx1–/–^ females by oral GTT (Figure 3E). Overall, no loss in β cell function or insulin content was seen in female Pex5^Pdx1–/–^ mice compared with controls (Figure 3, F–H), although females appeared to have a heterogenous phenotype in that elevations were observed in glucose- and KCl-dependent insulin secretion in 25% of the animals assessed (Figure 3, F and G).

### Reduced peroxisomal function in the pancreas of male mice has no impact on islet architecture or hormone content.

Enhanced GSIS in male mice that lack functional peroxisomes is not accounted for by changes in islet architecture or abundance of the islet hormones insulin and glucagon (Figure 4A). Furthermore, no changes were detected in pancreatic mass (Figure 4B) or β cell mass (Figure 4C) in Pex5^Pdx1–/–^ or Pex5^Ins1–/–^ mice compared with controls. These findings were confirmed with quantification of the insulin-positive area in the islet (Figure 4D) and islet fraction of the pancreas (Figure 4E). These data are consistent with islet insulin content being similar between genotypes (Figure 2, N and S). Additionally, no difference in circulating levels of insulin (Figure 4F) or glucagon (Figure 4G) were observed between genotypes.

### Increased oxidative phosphorylation pathway activity, palmitate oxidation, and ATP content in male mice with loss of peroxisomal function.

To identify differentially expressed genes (DEGs) that potentially contribute to altered β cell function in response to loss of peroxisomal function, mRNA abundance was analyzed by bulk RNA-Seq in isolated islets from male mice. We identified 3,270 DEGs between the genotypes (Figure 5A) with an enrichment in pathways involved in ribosome function, oxidative phosphorylation, lysosome function, and glycerolipid metabolism in the islets from Pex5^Pdx1–/–^ mice (Figure 5B). Several pathways involved in various aspects of inflammation, such as T cell receptor signaling, JAK/STAT, chemokine signaling, and cytokine–cytokine-receptor interaction pathways were downregulated in islets from Pex5^Pdx1–/–^ mice. Also, 20 genes involved in oxidative phosphorylation were upregulated in islets from Pex5^Pdx1–/–^ mice compared with controls (Figure 5C). However, the ratio of mitochondrial DNA to nuclear DNA was unchanged (Figure 5D), suggesting total mitochondrial number was similar between the genotypes. To determine whether the observed increase in GSIS in Pex5^Pdx1–/–^ mice may be due to a compensatory increase in long-chain fatty acid entry into mitochondria and subsequent oxidation, we assessed both pancreatic CPT1 activity and palmitate oxidation (Figure 5, E–G). Although CPT1 activity was similar between the genotypes (Figure 5E), complete oxidation of palmitate to CO_2_ was significantly increased in pancreatic mitochondria of Pex5^Pdx1–/–^ mice compared with controls (Figure 5F). This response was partially attenuated by both incubation with the CPT1 inhibitor etomoxir and addition of pyruvate (Figure 5F), suggesting that although CPT1 activity is not enhanced, it is required for the increase in palmitate oxidation in Pex5^Pdx1–/–^ mice. Incomplete oxidation of palmitate to acid soluble metabolites was similar between genotypes (Figure 5G). Oxidation of additional substrates including pyruvate (Figure 5H) and leucine (Figure 5I) were also unchanged between genotypes. Given that complete β-oxidation of palmitate generates substantial ATP through provision of acetyl-CoA to the TCA cycle and subsequent fueling of the electron transport chain and oxidative phosphorylation, we next quantified ATP levels. ATP content was significantly increased in islets from Pex5^Pdx1–/–^ mice incubated in media containing 11 mM and 20 mM glucose but not 2 mM glucose (Figure 5J). These data support findings of increased insulin secretion in response to glucose in male Pex5^Pdx1–/–^ mice (Figure 2). Similarly, ATP levels increased by 3.8-fold in Pex5-deficient 832/13 cells (Figure 5K). We interpret this data to indicate increased reliance on mitochondrial fatty acid oxidation, which produces more ATP per mol than glucose oxidation, in the peroxisome-compromised state.

### Reduction in CPT1a abundance in peroxisomal-deficient male mice restores β cell GSIS and islet ATP content to control levels.

Next, we simultaneously deleted Pex5 and Cpt1a in pancreatic tissue, thereby disrupting Cpt1a-mediated import of long-chain fatty acids into mitochondria in parallel with loss of peroxisomal function. Efficient and tissue-targeted gene deletion in Pex5/Cpt1a^Pdx1–/–^ mice was confirmed in islets by reduced *Pex5* and *Cpt1a* mRNA levels by RT-PCR, decreased Pex5 protein by Western blot, and reduced CPT1a protein abundance by IHC (Figure 6, A–C).

Insulin sensitivity was comparable in both males (Figure 6D) and females (not shown) between genotypes. Glucose tolerance at 14 and 26 weeks of age revealed significantly impaired glucose clearance in Pex5/Cpt1a^Pdx1–/–^ mice compared with controls (Figure 6, E and F). To evaluate β cell function, GSIS was analyzed 3 ways: (a) in vivo during a GTT (Figure 6G), (b) by hyperglycemic clamp (Figure 6, H–J), and (c) in isolated islets using perifusion (Figure 6, K and L). As previously shown, GSIS was elevated in Pex5^Pdx1–/–^ mice compared with controls (Figure 6G); however, GSIS in Pex5/Cpt1a^Pdx1–/–^ mice decreased in vivo compared with controls (Figure 6, G and J), with no difference between genotypes seen in isolated islets (Figure 6, K and L). During the hyperglycemic clamp, blood glucose levels were clamped at approximately 250 mg/dL for 120 minutes and were not significantly different between the genotypes (Figure 6H). The observed decrease in GSIS during a hyperglycemic clamp corresponds with a reduction in the glucose infusion rate in Pex5/Cpt1a^Pdx1–/–^ mice (Figure 6I). There were no differences in insulin content (Figure 6M), β cell transcription factor expression (Figure 6N), insulin-positive area (Figure 6O), islet fraction (Figure 6P), β cell mass (Figure 6Q), pancreas mass (Figure 6R), or circulating insulin levels (Figure 6S) across genotypes. Furthermore, unlike Pex5^Pdx1–/–^ mice (Figure 5), Pex5/Cpt1a^Pdx1–/–^ mice did not exhibit increased ATP levels at stimulatory glucose concentrations (Figure 6T). These findings highlight the indispensable role of mitochondrial fatty acid import and subsequent oxidation to provide ATP for enhanced GSIS under conditions of peroxisomal dysfunction in β cells.

### Loss of functional peroxisomes correlates with increased abundance of posttranslationally modified insulin 1 and 2 and a truncated insulin 2 peptide.

Despite increased GSIS in Pex5^Pdx1–/–^ mice, there was glucose intolerance with no change in peripheral insulin sensitivity. To uncover the mechanism underlying this contradictory phenotype, we performed liquid chromatography–ion mobility spectrometry–mass spectrometry (LC-IMS-MS) on islets from Pex5^CON^ and Pex5^Pdx1–/–^ mice. This technique is used to identify and quantify peptides with posttranslational modifications and can also identify fragmented peptides. Retention time of insulin 1 (green box) and 2 (orange box) by LC chromatogram (Figure 7A) and the entire mass spectrum revealing all insulin 1 and 2 peaks at different charge states and the arrival time distribution of peaks at 966.4 *m/z* and 1,159.5 *m/z* (Figure 7B) were determined. The relative ion abundances of both insulin 1 and insulin 2 showed upward trends in Pex5^Pdx1–/–^ mice compared with controls, though these increases were not statistically significant (Figure 7B). The ion abundance of oxidized insulin 1 (Figure 7, C and F) and insulin 2 (Figure 7, D and H) was markedly increased in islets from Pex5^Pdx1–/–^ mice compared with controls. Specifically, the proportion of insulin 1 detected in an oxidized state increased by 102%, rising from 4.6% in Pex5^CON^ mice to 9.3% in Pex5^Pdx1–/–^ mice (Figure 7G). Similarly, oxidized insulin 2 increased by 54%, from 8.5% in controls to 13.1% in islets from Pex5^Pdx1–/–^ mice (Figure 7I). Furthermore, a truncated insulin 2–derived peptide, detection of which was almost negligible in islets from Pex5^CON^ mice, reached nearly 2% of total insulin 2 in islets from Pex5^Pdx1–/–^ mice (Figure 7, E, J, and K). Combined with findings of elevated GSIS and concomitant reductions in glucose tolerance, these data suggest that oxidative modifications to the insulin protein may limit its function in circulation.

### Coordinated alterations in oxidative stress signature in pancreas and islets from Pex5^Pdx1–/–^ mice.

Metabolomics analyses were performed on pancreas from 12-week-old and 30-week-old males, as well as 36-week-old females, corresponding to ages when GTTs and GSIS assays were performed (Figure 2), to identify metabolites associated with changes in insulin oxidation and secretion. We detected189 metabolites (Figure 8, A–C, and Supplemental Figure 4); the majority of significantly altered compounds were observed in 12-week-old males (27 metabolites; 12 upregulated and 15 downregulated in Pex5^Pdx1–/–^ mice) and 30-week-old males (34 downregulated metabolites in Pex5^Pdx1–/–^ mice), and only 2 upregulated metabolites were detected in female Pex5^Pdx1–/–^ mice. Most notable were increased abundance of glutathione (Figure 8A), a major intracellular antioxidant (14); 8-hydroxy-2′-deoxyguanosine (Figure 8, A and D), an oxidative DNA modification (15); and 4-hydroxynonenal (Figure 8, A and E), a downstream byproduct of lipid peroxidation (16), in 12-week-old Pex5^Pdx1–/–^ mice. Interestingly, most of these metabolites were either downregulated or unchanged between genotypes in older Pex5^Pdx1–/–^ mice (Figure 8B and Supplemental Figure 4). These findings suggest an early increase in oxidative stress in β cells of Pex5^Pdx1–/–^ male mice correlating with alterations in β cell function and glucose homeostasis (Figure 2).

To directly investigate markers of oxidative stress, we measured lipid peroxidation using electron paramagnetic resonance (EPR) spectroscopy. The 6-line spectrum in Figure 8F is consistent with POBN-LOO^•^, a lipid peroxyl radical trapped by the chemical compound POBN (identified through hyperfine coupling constants a^N^ = 15.75 ± 0.06 G and *a_β_*^H^ = 2.77 ± 0.07 G) (17). EPR signal intensities of the measured peak amplitudes confirmed an 80.8% increase in lipid peroxidation in Pex5^Pdx1–/–^ mice (Figure 8G). Further, we observed an 81.3% increase in protein carbonylation, an irreversible protein modification indicative of oxidative damage (18), in islets from male Pex5^Pdx1–/–^ mice compared with controls (Figure 8H). RNA-Seq analysis revealed several key genes that participate in cellular antioxidant defenses were upregulated in islets from Pex5^Pdx1–/–^ mice, including *Sod1* (Figure 8I) and *Gpx1* (Figure 8J). These data support a model of increased oxidative stress in the absence of functional peroxisomes driving oxidative modifications of DNA, lipids, and proteins including insulin in the β cell.

### Global shifts in transcriptional signature in islets from Pex5^Pdx1–/–^ mice.

RNA-Seq analysis uncovered that peroxisomal insufficiency in the pancreas produced a gene signature associated with β cell immaturity or transdifferentiation. Specifically, gene expression patterns in islets indicated a shift toward an exocrine pancreas-like (acinar cell) phenotype. In addition to a decrease in markers of β cell maturity (e.g., *Mafa*, *Mafb*), *Npy*, a marker of immature β cells, was upregulated in islets of Pex5^Pdx1–/–^ mice (Figure 9A). Furthermore, expression of genes more commonly expressed in exocrine pancreatic tissue were upregulated in islets from Pex5^Pdx1–/–^ mice including proteases (e.g., *Cela1*, *Cpa1*, *Prss2*, *Spink1*, and *Try4*), lipases (e.g., *Cel*, *Clps*, *Pnlip*, *Pnliprp1*, and *Pnliprp2*), and genes encoding regulatory factors (e.g., *Reg1*, *Reg3a*, and *Rnase1*) (Figure 9A).

To confirm that loss of β cell maturity markers and dedifferentiation occur in response to decreased peroxisomal function, we stained pancreatic tissue for Nkx6.1 and ALDH1A3. Nkx6.1 is a transcription factor that regulates insulin gene expression, and decreased nuclear staining of Nkx6.1 is associated with a decrease in β cell maturity (19). Decreased nuclear abundance of Nkx6.1 was observed at 15 and 30 weeks of age in Pex5^Pdx1–/–^ mice compared with controls (Figure 9B). ALDH1A3 is a marker of β cell dedifferentiation in human (20, 21) and mouse models of type 2 diabetes (22, 23) and is also upregulated in other models of β cell dysfunction (7, 24–26). We noted increased colocalization of ALDH1A3 with insulin in islets at 6 weeks of age in Pex5^Pdx1–/–^ mice that progressively worsened with age (Figure 9C). A similar phenotype was seen in Pex5^Ins1–/–^ mice on a more delayed timeframe, with widespread detection of ALDH1A3 in islets at 52 weeks (Figure 9D).

### Loss of functional peroxisomes in pancreatic tissue promotes β cell dedifferentiation in female mice.

RNA-Seq analysis from female islets showed only 18 DEGs (Figure 10A), indicating a sexually dimorphic transcriptional signature in Pex5^Pdx1–/–^ mice consistent with the milder metabolic phenotype compared with male mice (Figure 3). Two genes were confirmed by RT-PCR to be upregulated in islets from female Pex5^Pdx1–/–^ mice: *Npy*, also increased in male mice, and *Nptx2* (Figure 10B). Neuropeptide Y (*Npy*) is expressed in progenitor cell populations during pancreatic development and epigenetically downregulated in mature β cells (27). In addition, NPY expression increases in β cells in humans and mice with type 2 diabetes and correlates with reduced β cell function (27, 28). Furthermore, ALDH1A3 was notably expressed in β cells of Pex5^Pdx1–/–^ mice (Figure 10C).

However, unlike islets from male Pex5^Pdx1–/–^ mice that displayed increased expression of genes involved in glycerolipid and oxidative phosphorylation metabolism (Figure 5, B and C), KEGG analysis in islets from female Pex5^Pdx1–/–^ mice showed an overall decrease in the glycerolipid metabolism pathway (Figure 10, D and E), with no evidence of altered expression of genes regulating oxidative phosphorylation (Figure 10D). Similarly, acinar-enriched genes that were upregulated in islets from male Pex5^Pdx1–/–^ mice were either reduced in females (Spink1, Clps, Cpa1, Cela3b, Prss2, and Reg3g; Figure 10A) or showed no change (Pnliprp1, Reg1, and Reg3a; Figure 10, F–H). Overall, the RNA-Seq datasets were consistent with the premise that peroxisomal insufficiency in the pancreas drives β cell immaturity and dedifferentiation, although the phenotype was more severe in male mice. Another key finding revealed here is that male mice had a robust transcriptomic shift toward an acinar-like phenotype and metabolic reprogramming, whereas females displayed a more limited loss of β cell identity markers.

## Discussion

The pancreatic islet β cell senses various nutrients and responds with appropriate output based on metabolic signals, with the classic example being glucose metabolism coupled to insulin secretion (29). In addition, the depletion of fatty acids (30) or the restriction of fatty acid import into mitochondria (7) decreases GSIS. Thus, there is clear interplay between discrete fuel stimuli necessary to sustain appropriate insulin secretion events. One organelle involved in lipid sensing and processing, including very-long-chain fatty acid metabolism and synthesis of various lipid species, is the peroxisome.

A previous report suggested that reduced peroxisomal function impairs GSIS (4). However, several aspects of the previous study design may explain the phenotypic differences observed. The Pex5 floxed mouse used by Baes et al. is on a Swiss background (31), whereas Pex5 floxed mice in our studies are on a C57BL/6J background. The previous study used the RIP-Cre (Ins2-Cre) driver line, which has been shown to have impaired glucose tolerance and decreased GSIS (32). The RIP-Cre also exhibits ectopic expression across multiple brain regions, as evidenced by its recent use for driving “specific hypothalamic deletion” (33) and is no longer considered a β cell–specific Cre. Despite the known limitations, no Cre-only control was included in that study. Therefore, decreased GSIS in their model may be due to issues with Cre expression, rather than targeted KO of the Pex5 gene.

In the present study, our genetic tools enabled a more rigorous evaluation of peroxisomes in supporting β cell function and maturity. We primarily employed the Pdx1-Cre line, which targets both exocrine and endocrine cells; however, exocrine cells exhibited substantially lower peroxisome abundance when compared with β cells within the endocrine pancreas (Figure 1D). Key findings were replicated in the β cell–restricted Ins1-Cre model. Several major outcomes were further confirmed in 832/13 cells, with phenotypes consistent across all models. Confirmation of similar outcomes in 832/13 cells strongly argues against confounding developmental effects arising from constitutive Cre-mediated deletion. Therefore, unlike the previous report, our rigorous in vivo and ex vivo approaches provide strong evidence for a β cell–intrinsic mechanism.

In male mice, we found that enhanced GSIS in Pex5^Pdx1–/–^ mice correlated with enhanced mitochondrial β-oxidation of palmitate, an upregulation of oxidative phosphorylation pathways genes, and elevated islet ATP levels. These findings are consistent with fatty acid requirements for optimal GSIS (6, 7, 30). Despite the increased mitochondrial lipid oxidation observed, CPT1 activity remained unchanged. This observation supports previous findings, in which a 30% rise in [^3^H]palmitate oxidation was observed in isolated islets from Harlan Sprague-Dawley rats fed a 60% fat diet versus 4% fat diet, without corresponding changes in CPT1 gene expression (34). These data emphasize that the β-oxidative machinery of islets is sufficiently robust to accommodate increased lipid flux without requiring upregulation of CPT1 or related enzymes (34). Our findings align with these observations, suggesting that β cells can rapidly augment fatty acid oxidation to support elevations in insulin secretion without the need for increased CPT1 expression or activity.

To confirm that increased GSIS associated with peroxisomal dysfunction required mitochondrial fatty acid transport, a double KO lacking both the Pex5 and Cpt1a genes was generated. This unique model combined peroxisomal dysfunction with impaired mitochondrial import of long-chain fatty acids. Reduction of mitochondrial Cpt1a on a Pex5 deletion background abolished both the increase in ATP content and GSIS at stimulatory glucose concentrations, demonstrating a requirement for augmented mitochondrial lipid oxidation in the presence of dysfunctional peroxisomes to enhance insulin secretion. Although GSIS increases during peroxisomal dysfunction, the increase in β cell function is paradoxically linked to impaired glucose tolerance. In the absence of systemic insulin resistance, a more detailed analysis of insulin integrity showed that, in Pex5^Pdx1–/–^ islets, a total of 22% of all detected insulin species are oxidized, compared with only 13% combined in control islets. These data strongly suggest oxidative stress in the absence of functional peroxisomes. Additionally, an insulin 2 peptide was detected, indicating further posttranslational disruption of hormone integrity when peroxisomal function was impaired.

Oxidative stress, arising from an imbalance between ROS generation and the cellular antioxidant defense systems, promotes covalent modification of proteins, lipids, carbohydrates, and nucleic acids (35). Peroxisomes provide essential antioxidant mechanisms to preserve redox homeostasis (36). We observed an increase in cellular oxidative stress in Pex5^Pdx1–/–^ mice, including increased lipid peroxidation and protein carbonylation (Figure 8). Oxidative modification of proteins occurs either directly via ROS or indirectly through secondary byproducts of oxidative stress, including lipid peroxidation products such as 4-HNE, and is associated with aging, cancer, cardiovascular disease, and neurodegenerative disorders. These modifications lead to structural and functional alterations including subunit dissociation, protein unfolding, backbone fragmentation, and the formation of nondegradable protein aggregates; these modifications often impair or abolish protein function, thereby necessitating clearance through the proteasomal degradation system (37–41). Accordingly, the formation of oxidized proteins, protein fragments, and/or aggregates may contribute to the increased expression of proteasomal pathway genes observed in Pex5^Pdx1–/–^ islets. We also observed upregulation of lysosomal pathway components in Pex5^Pdx1–/–^ islets, and elevated LAMP2 staining, an indicator of lysosomal activation, was detected in Pex5^RIP–/–^ mice (4).

However, the specific causes and functional consequences of insulin oxidation in vivo remain poorly defined. In vitro exposure of human insulin to ROS induced oxidative modifications that reduced its biological activity (42). Consistent with this finding, incubation of insulin with plasma from patients with diabetes increased the detection of oxidation products and correlated with reduced hormone activity in subsequent i.p. ITTs (43).

Cells have evolved multiple, coordinated lines of defense to maintain ROS within a physiological range, thereby limiting oxidative damage to DNA, proteins, and lipids and preserving cellular integrity (44–46). The cellular redox state is principally defined by the ratio of reduced (GSH) to oxidized (GSSG) glutathione. GSH, which plays a central role in antioxidant defense by facilitating the conversion of hydrogen peroxide and lipid peroxides into less reactive species (14), was increased in 12-week-old Pex5^Pdx1–/–^ mice. We further found that the expression of the *Sod1* and *GPx4* genes was increased in islets of Pex5^Pdx1–/–^ mice. Another protective mechanism involves detoxification of reactive lipid aldehydes (e.g., 4-HNE found to be increased in 12-week-old Pex5^Pdx1–/–^ mice) by phase I and II metabolic enzymes, with phase I metabolic enzymes including ALDH using the NAD(P)^+^/NAD(P)H cofactors. Finally, oxidatively modified proteins that cannot be repaired are targeted for degradation, representing a last line of defense to preserve cellular integrity.

In islets from Pex5^Pdx1–/–^ mice, it remains unclear whether the truncated insulin peptides detected by IMS-MS are actively targeted for degradation via the proteasome due to oxidative damage, or whether they are cleaved through pathways independent of proteasomal degradation. One candidate for such processing is the insulin-degrading enzyme (IDE) (30), which has exceptionally high affinity for insulin. Several of its known cleavage products feature intact A chains with cleavage at the B24-B25 and B25-B26 peptide bonds (47), consistent with the fragment we identified by IMS-MS. Notably, IDE activity is influenced by environmental factors, including oxidative stress, and glutathione enhances IDE-mediated insulin degradation (48).

In contrast to the pronounced hypersecretory phenotype observed in male mice with peroxisomal dysfunction, the phenotype in females is comparatively mild. Although female Pex5^Pdx1–/–^ mice exhibit modest glucose intolerance, there is no overall change in β cell function. Moreover, female Pex5^Ins1–/–^ mice maintain normal glucose tolerance. Unlike in males, RNA-Seq in females did not reveal upregulation of genes involved in the oxidative phosphorylation pathway, suggesting that the mitochondrial compensatory mechanisms observed in males may be absent or blunted in females. These findings raise 3 possibilities: (a) the progression of β cell dysfunction may be delayed in females, and a more pronounced phenotype might emerge at later time points (e.g., 1 year vs. 36 weeks of age); (b) female mice may exhibit a protective mechanism linked to elevated carnitine levels observed by metabolomic profiling, consistent with the known antioxidant properties of this metabolite (49); or (c) estrogen is protective against oxidative stress, reducing the severity of the peroxisomal loss of function in female mice. This latter notion is consistent with known effects of estrogen on isolated organelles (50).

Despite the absence of overt GSIS abnormalities or severe glucose intolerance in females, both male and female Pex5^Pdx1–/–^ mice show evidence of reduced β cell maturity. Notably, ALDH1A3 was also upregulated in female Pex5^Pdx1–/–^ mice. ALDH1A3 has been identified as a marker of β cell dedifferentiation in both human pancreatic tissue and mouse models of type 2 diabetes (20-23). However, as noted above, ALDH enzymes also play a critical role in the detoxification of reactive lipid aldehydes such as 4-HNE. Interestingly, ALDH1A3-deficient human glioblastoma cells exhibited significantly elevated levels of 4-HNE–modified proteins compared with WT controls (51), underscoring a protective role for ALDH1A3 in mitigating aldehyde-induced oxidative damage. These findings are in line with the possibility that ALDH1A3 is upregulated in peroxisome-deficient β cells as a protective response against oxidative damage and may explain the progressive, age-dependent increase in ALDH1A3 expression observed in Pex5^Pdx1–/–^ mice, along with the decreased 4-HNE abundance in older males.

In conclusion, mice lacking functional peroxisomes exhibit 3 distinct impairments in β cell function: (a) elevated GSIS, (b) oxidation/truncation of the insulin protein, and (c) loss of β cell maturity/identity markers. However, several ambiguities remain, including whether these phenotypes are mechanistically independent or whether a common underlying pathway is engaged.

## Methods

### Sex as a biological variable.

Male and female mice were used in this study, and sex-dimorphic effects are reported.

### Cell culture and reagents.

The 832/13 rat insulinoma cells (a gift from Chris Newgard, Duke University Medical Center, Durham, North Carolina) were cultured as described previously (52). Mycoplasma-free status was confirmed using the MycoAlert mycoplasma detection kit (Lonza). Negative control (51-01-14-04) and Pex5-targeted DsiRNAs (design IDs: rn.Ri.Pex5.13.1 and rn.Ri.Pex5.13.2) were from Integrated DNA Technologies and transfected using DharmaFECT Transfection Reagent 1 (Thermo Fisher Scientific) per the manufacturer’s instructions. For GSIS assays, 832/13 cells were treated as previously described (52). Secreted insulin was measured using the High Range Rat Insulin ELISA kit (Mercodia), cells were lysed using M-PER lysis reagent (Thermo Fisher Scientific), and total protein quantified by BCA assay (Thermo Fisher Scientific) to normalize insulin secretion to cellular protein content. For ATP measurements, cells and islets (50 per genotype) were treated identically to GSIS conditions. ATP content was measured using the ATP Bioluminescence assay kit (MilliporeSigma) as previously described (7).

### Experimental animals.

The C57BL/6J (stock 000664), Pdx1-Cre (stock 01647), Ins1-Cre (stock 026801), and Pex5^fl/fl^ (stock 031665) mice were purchased from The Jackson Laboratory. Pancreas-specific Pex5 gene deletion mice (Pex5^Pdx–/–^) were generated by crossing female Pex5^fl/fl^ mice with male Pdx1-Cre mice. Mice with a β cell–specific deletion of Pex5 (Pex5^Ins1–/–^) were generated by crossing female Pex5^fl/fl^ mice with male Ins1-Cre mice. Cpt1a^fl/fl^ mice were obtained from Taconic (C57BL/6-*Cpt1a^tm1.1mrl^* model 9759), and Cpt1a^Pdx1–/–^ mice have been previously described (7). Mice were group-housed with a 12-hour light/12-hour dark cycle at 22 ± 2°C with ad libitum access to Rodent Diet 5015 (LabDiet, 20% protein, 26% fat, and 54% carbohydrates) and water. Body composition was measured by NMR using Bruker Minispec LF110 and LF50 Time-Domain systems. Energy expenditure, respiratory quotient, activity, and caloric intake were assessed using Promethion metabolic cages (Sable Systems). Mice were single-housed and acclimated to training cages for 1 week prior to metabolic cage measurements. We bred 4–5 independent cohorts of mice (both Pdx1-Cre and Ins1-Cre). After completion, mice were euthanized by CO_2_ asphyxiation followed by decapitation after a fasting period of 4 hours. Trunk blood was collected, and tissues were snap-frozen. Pancreata were either snap-frozen or fixed in 10% neutral-buffered formalin. Islets were isolated following our standard laboratory procedure (53).

### GTTs, ITTs, and in vivo GSIS.

GTTs and ITTs were performed as previously described (7). For oral GTTs, mice were gavage trained for 3 days prior to administration of glucose. For measurements of serum insulin during GTT (i.p.), blood was collected from the tail vein at time points shown in figure legends. Blood was subsequently spun at 4°C at 7,200*g* for 20 minutes, and the serum fraction was extracted.

### Hyperglycemic clamp.

Catheter cannulation was conducted in 18-week-old mice as previously described (54–56). The left carotid artery was cannulated for sampling and the right jugular vein for glucose infusion. Catheter patency was maintained via heparinized glycerol (500 U/mL). One week after cannulation, mice were fasted in clamp cages for 5 hours (8:00 am–1:00 pm). Catheters were externalized 1 hour prior to the infusion starting time, flushed with heparinized saline (20 U/mL), and connected to microsyringe pumps with swivels allowing mice to move freely throughout the procedure. Blood glucose levels were sampled every 10 minutes during infusion using Ascensia Contour glucometers. Glycemia was maintained at approximately 250 mg/dL by adjusting infusion rates of 50% glucose solution as detailed in Figure 2G and Figure 6H. Plasma samples were collected at the time points of 0, 10, 20, 30, 70, 80, 110, and 120 minutes for insulin measurement by insulin ELISA kit from Mercodia. After collecting plasma samples, blood cells were returned to each mouse via the arterial catheter.

### Islet isolation and islet perifusion.

Pancreatic islets were isolated from mice shipped to Vanderbilt University Medical Center following an overnight quarantine in the Translational Pathology Shared Resource using the protocol (https://www.protocols.io/view/mouse-pancreatic-islet-isolation-bp2l6boe5gqe/v1). Isolated islets underwent functional assessment within 2 hours after isolation (https://www.protocols.io/view/mouse-islet-perifusion-3-stimuli-protocol-n2bvj6yenlk5/v1). Insulin concentration in islet extracts and perifusion fractions was measured by ELISA (Mercodia). Results were normalized to islet equivalents.

### Immunoblotting.

Frozen pancreatic tissue (50 mg) was homogenized in 300 mL T-PER lysis reagent (Thermo Fisher Scientific) supplemented with Halt Protease Inhibitor Cocktail (Thermo Fisher Scientific), and 100–150 islets per mouse were isolated and lysed in 50 mL of T-PER with Halt Protease Inhibitor Cocktail. Protein was quantified using a BCA assay. A detailed protocol with our standard reagents has been published previously (57). Primary antibodies used were from Cell Signaling Technology: Pex5 (CST-83020) and β-actin (CST-3700).

### RNA isolation, cDNA synthesis, and gene expression analysis.

Total RNA was isolated from 30 mg of frozen tissue or isolated islets (100–150 islets per mouse), using the RNeasy Mini RNA kit from QIAGEN. Procedures for cDNA synthesis, primer design, and RT-PCR were performed using our standard laboratory protocol (58).

### Metabolomics.

Pancreatic tissue samples (100 mg) were analyzed at the Biological Mass Spectrometry Core at the University of Tennessee (RRID: SCR 021368). Metabolites were extracted following a previously published untargeted metabolomics protocol for water-soluble compounds (59). Briefly, extraction was performed using a solvent mixture consisting of methanol, acetonitrile, and water (2:2:1, v/v/v) containing 0.1 M formic acid at 4°C. Samples were centrifuged at 21,300*g*, and supernatants were dried under nitrogen gas. Extracts were reconstituted in 300 μL of water for ultra-HPLC–high-resolution MS (UHPLC–HRMS) analysis.

Metabolomic profiling was performed using UHPLC coupled to high-resolution Orbitrap MS following a previously established method (60). Briefly, water-soluble metabolites were separated on a Synergi Hydro-RP column (2.5 μm particle size, 100 Å pore size, 100 × 2.0 mm; Phenomenex) using a reverse-phase gradient with tributylamine as the ion-pairing agent and analyzed on an Exactive Plus Orbitrap mass spectrometer (Thermo Fisher Scientific) operated in negative electrospray ionization mode. Full-scan mass spectra were acquired over an *m/z* range of 72–1,000 at a resolving power of 140,000 (at 200 *m/z*).

Raw mass spectral data files generated using Xcalibur were converted to the mzML format using the open-source MsConvert utility within the ProteoWizard framework (61). The resulting mzML files were imported into the Metabolomic Analysis and Visualization Engine (MAVEN) for downstream processing (62, 63). MAVEN was used to perform peak alignment and correct for retention time variability across samples. Metabolites were manually integrated based on exact mass accuracy (± 5 ppm) and retention time matching (± 1.5 minutes) relative to an in-house reference library containing more than 300 authenticated metabolite standards and validated with isotopic abundance patterns (using relative isotope spacing, Δ*m/z* ± 0.0002 Da). Before downstream analysis, raw metabolite intensity data were first normalized by weight to account for differences in overall signal intensity between samples. Normalized metabolite data were imported into R for statistical analysis and generation of plots. Partial least squares discriminant analysis (PLS-DA; Supplemental Figure 4, A–C) was performed using the mixOmics package (64). Heatmaps were generated with the package ComplexHeatmap (65). Volcano plots were generated with the package EnhancedVolcano using the *P* values calculated from the default t.test function in R (66).

### Lipidomics.

Pancreatic tissue samples (100 mg) were used for lipid extractions using a modified Bligh and Dyer extraction protocol (67). Briefly, 750 μL of 1:2 chloroform/methanol, 250 μL of chloroform, and 250 μL of water were added to each sample. Organic layer from each sample was collected, then dried under nitrogen and resuspended in 300 μL of 9:1 methanol/chloroform. Before UHPLC-HRMS analysis, 30 μL of each resuspended sample was diluted in 270 μL of acetonitrile.

Ether lipids profiling was achieved using reverse-phase chromatography, which separated the lipid species based on their acyl chain composition using a C30 RP column (68). Analyses were conducted using a Vanquish UPLC system (Thermo Fisher Scientific) coupled to an Orbitrap Exploris Tribrid 120 mass spectrometer (Thermo Fisher Scientific) operated with a heated electrospray ionization source in positive and negative ionization modes. Data were acquired in data-dependent acquisition mode with full-scan MS^1^ spectra (*m/z* 150–2,000) collected at 120,000 resolution (at *m/z* 200) and ddMS^2^ spectra acquired at 30,000 resolution (at *m/z* 200) using stepped collision energies of 20, 50, and 75 eV.

Raw mass spectral data files for positive and negative mode generated from Xcalibur were converted to the analysis base file (ABF) format using the open-source ABF converter. The resulting ABF files were imported into MS-Dial for downstream processing (69). MS-Dial was used to perform peak alignment and correct for retention time variability across samples. Ether lipid peaks were manually integrated based on retention times (± 2 minutes), exact mass accuracy (± 10 ppm), and fragmentation spectra matching (low score, high score, or no MS2 match) relative to an in-built in silico library (MSI level 2) (70). Before downstream analysis, the raw intensity data for annotated lipid peaks generated from MS-Dial were also normalized by the mass of each sample used for extraction. Normalized lipid data were imported into R for statistical analysis and generation of plots. Volcano plots were generated with the package EnhancedVolcano using the *P* values calculated from the default t.test function in R (66).

### Single islet analysis with LC-IMS-MS.

Islets from Pex5^CON^ and Pex5^Pdx1–/–^ mice were transferred into 150 μL of 5% methanol containing 0.1% formic acid for peptide extraction. Samples were centrifuged at 1,500*g* for 3 minutes, and the supernatants were collected for LC-IMS-MS analysis.

Peptide analyses were performed using an Agilent 1260 Infinity LC system coupled to an Agilent 6560 IMS-QTOF mass spectrometer (Agilent Technologies) as previously described (71, 72). Mobile phase A (MPA) consisted of 0.1% formic acid in LC-MS–grade water, and mobile phase B (MPB) consisted of acetonitrile. A 5 μL injection volume was used with a flow rate of 200 μL/min. The following gradient was applied: 98% MPA (0–3 minutes), 80% MPA (3–5 minutes), 65% MPA (5–18 minutes), 50% MPA (18–23 minutes), 25% MPA (23–26 minutes), 10% MPA (26–31 minutes), and 5% MPA (31–33 minutes). The column was held at 5% MPA for 2 minutes, then re-equilibrated to 98% MPA from 35 to 40 minutes and maintained for an additional 5 minutes. Chromatographic separation was achieved using a Zorbax Extended C18 column (2.1 × 50 mm; Agilent Technologies) maintained at 40°C. Eluted peptides were ionized using a jet stream electrospray ionization source and introduced into a nitrogen-filled drift cell (3.95 torr) for single-field ion mobility analysis at an entrance voltage of 1,600 V. Ions were guided through a hexapole ion guide into the QTOF analyzer, and data were acquired in positive ion mode. Each biological group included 4 biological replicates (*n* = 4 per genotype), and samples were analyzed in technical triplicate.

### Serial analysis of gene expression, gene expression data analysis, and pathway enrichment analysis.

RNA was isolated from 22-week-old male and 36-week-old female Pex5^CON^ and Pex5^Pdx1–/–^ mouse islets (100–150 islets per mouse). Construction of sequencing libraries and differential gene expression analysis were as described (7). Sequencing was performed on 4 biological replicates per genotype from male mice and 5 biological replicates per genotype from female mice. Principal component analysis identified 1 male Pex5^Pdx1–/–^ and 1 female sample per genotype as outliers, which were removed from subsequent analysis. After outlier removal, genes with at least 1 count per million (CPM) reads in 3 or more samples were retained for further analysis, resulting in 13,221 genes in males and 12,558 genes in females. To adjust for multiple testing, genes with an adjusted *P* value less than 0.05 were considered to be differentially expressed.

Pathway enrichment was conducted via the competitive gene-scoring based gene set enrichment analysis software (GSEA) (73). GSEA was performed via the preranked option (73) by first ranking all genes by their log fold-change estimates and then using a Kolmogorov-Smirnov test to determine whether the gene ranks deviated significantly from a uniform distribution in a priori defined KEGG gene sets obtained from MSigDB (74). Statistical significance of the observed enrichment was assessed by permutation testing over size-matched gene sets. Significant gene sets were selected by control of FDR at 5% or less (75).

### Pancreas IHC.

Our standard methods for pancreas fixing, embedding, sectioning, quantification of insulin-positive area, islet fraction, β cell mass, and IHC detection of Cpt1a have been described previously (7). For immunofluorescence, primary antibodies were the following: guinea pig anti-insulin (DAKO, IR004, 1:200), rabbit anti-ALDH1A3 (Novus Biologicals, NBP2-15339, 1:200), mouse anti-Nkx6.1 (Cell Signaling Technology, 54551, 1:400), mouse anti-glucagon (Cell Signaling Technology, 8233, 1:400), rabbit anti-Acox1 (Abcam, ab184032, 1:250), and mouse anti-PMP70 (MilliporeSigma, SAB4200181, 1:500). FFPE sections were dewaxed and subjected to heat-induced epitope retrieval (20 minutes at 100°C, Biocare Medical Decloaking Chamber) with a pH 6.0 citrate buffer (H-3300, Vector Laboratories). Sections were photobleached in a solution containing PBS, 30% H_2_O_2_, and 1 M NaOH for 1 hour. Primary antibodies were diluted in Leica Bond Diluent and incubated overnight at 4°C, followed by three 5-minute washes in TBS with Tween 20 (TBST) and a 2-hour incubation with fluorescently-conjugated secondary antibodies (1:300) from Invitrogen: goat anti-guinea pig (Alexa Fluor 48, 11073), goat anti-rabbit (Alexa Fluor 647, 32733), goat anti-rabbit (Alexa Fluor 555, A32732), and goat anti-mouse IgG1 (Alexa Fluor 647, A21240). Slides were washed 3 times in TBST, counterstained with Hoechst 33342 (Invitrogen, H1399) for 10 minutes, washed once each in TBST and H_2_O, and mounted with Aqua-Mount (Epredia, 13800). Slides were imaged on a Zeiss Axioscan 7 microscope, Leica SP5, or a Leica STELLARIS confocal using a HC PL APO 93x/1.30 GLYC motCORR STED objective. For high-resolution images (Figure 1, D and E), maximum-intensity projections were composed of 25 slices taken at 0.33 mm steps.

### Serum analyses and triglyceride determination.

Serum insulin and glucagon were measured using the mouse insulin and glucagon ELISA kits from Mercodia. Triglyceride (serum and pancreas) was calculated using the serum triglyceride determination kit from Sigma-Aldrich with minor protocol modifications as described recently (7).

### In vivo spin trapping and EPR spectroscopy.

The compound α-(4-pyridyl-1-oxide)-N-t-butyl nitrone (POBN) was used for spin-trapping lipid radicals; POBN was dissolved in saline and administered i.p. at 500 mg/kg body weight. Pancreas tissue samples from each experimental group were collected 45 minutes after injection, immediately frozen in liquid nitrogen, and stored at –80°C until EPR measurements. Lipid extraction was performed using chloroform/methanol (2/1) (Folch-extraction) as described previously (17). All EPR spectra were recorded in a quartz flat cell using an X-band EMX plus EPR spectroscope (parameters: 3,480 ± 80 G scan width, 10^5^ receiver gain, and 20 mW microwave power; time constant: 1,310 ms; conversion time: 655 ms). EPR spectra were analyzed using Bruker WinEPR software, measuring the amplitude of the 6-line signal, expressed as arbitrary units. Data were normalized to 300 mg tissue.

### Protein carbonylation.

Protein carbonyl content was quantified using the protein carbonyl ELISA kit from Abcam with modifications to the manufacturer’s protocol. Briefly, islets were isolated from 4–6 mice per genotype, lysed by sonication (Bioruptor Pico, Diagenode), followed by nuclease removal using 0.05% polyethylenimine (Millipore Sigma). Protein concentration was determined by BCA assay, and samples were diluted to an empirically determined concentration of 40 μg/mL.

### Data availability.

The RNA-Seq dataset is in NCBI’s Gene Expression Omnibus (GEO GSE308937). The source data underlying all graphs and reported means are provided in the Supporting Data Values file, with individual tabs corresponding to each figure panel.

### Statistics.

Data are shown as mean ± SEM. Unless otherwise stated above, statistical analysis was performed using GraphPad Prism (v10.4.1). Unless stated otherwise in the figure legend, all data were analyzed by two-tailed Student’s t-test, one-way analysis of variance (ANOVA) with multiple comparisons, or multiple unpaired t tests.

### Study approval.

All procedures involving mice were approved by the IACUCs of Pennington Biomedical Research Center, Vanderbilt University Medical Center, The University of Tennessee Graduate School of Medicine, and The University of Alabama at Birmingham.

## Author contributions

JJC and SJB conceptualized the study and wrote the manuscript. CRC, MPD, MAL, RCN, DHB, SDD, MDK, KS, SSH, TDD, AC, MB, TK, KMH, ZAV, QS, and SJB performed experiments. JJC, CRC, MPD, TMM, MAL, RCN, DHB, SDD, MDK, KS, SSH, TDD, AC, MB, TK, KMH, SG, ZAV, QS, SRC, and SJB analyzed data. JJC, CRC, MPD, TMM, MAL, RCN, DHB, SDD, MDK, KS, SSH, TDD, AC, MB, TK, KMH, SG, ZAV, QS, SRC, and SJB reviewed the manuscript.

## Conflict of interest

The authors have declared that no conflict of interest exists.

## Funding support

This work is the result of NIH funding, in whole or in part, and is subject to the NIH Public Access Policy. Through acceptance of this federal funding, the NIH has been given a right to make the work publicly available in PubMed Central.

- NIH R56 DK141177-01 (to JJC and RCN).
- NIH R01 DK123183-05 (to JJC).
- NIH R01 DK137472 (to KS).
- NIH P20 GM135002 (to SJB).
- NIH T32DK064584 (to MAL).
- NIH T32AT004094 (to TMM).
- NIH P30 DK072476.
- NIH S10 OD02370.

## Supplementary Material

Supplemental data

Unedited blot and gel images

Supporting data values

## Figures and Tables

**Figure 1:**
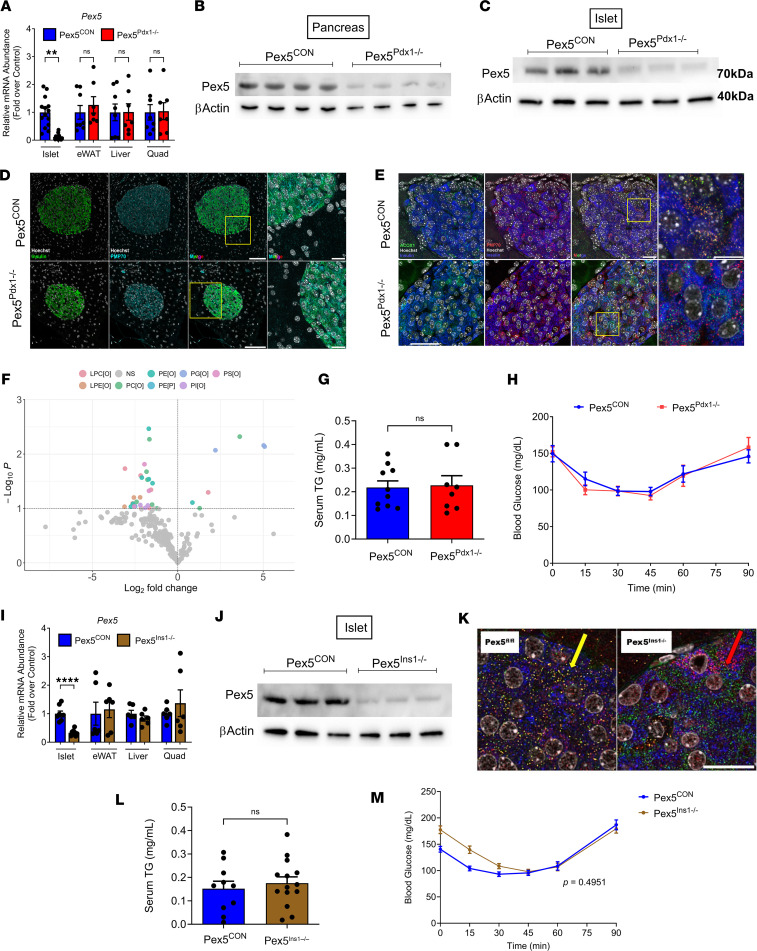
Pancreas- and β cell–specific deletion of Pex5 impairs peroxisomal matrix protein import and function. (**A**) *Pex5* mRNA abundance by RT-PCR from Pex5^CON^ and Pex5^Pdx1–/–^ mice; *n* = 7–14. (**B**) Pex5 abundance by immunoblot in pancreatic lysates and (**C**) isolated islets; *n* = 3–4. (**D**) Pancreatic tissue from Pex5^CON^ (top panels) and Pex5^Pdx1–/–^ (bottom panels) mice costained for PMP70 (cyan), insulin (green), and Hoechst (gray); *n* = 3 with representative image shown. Scale bars:100 μm (columns 1–3) and 20 μm (last column is a higher-resolution image of tissue denoted by yellow box in 3rd column). (**E**) Pancreatic tissue costained for Acox1 (green), PMP70 (red), insulin (blue), and Hoechst (gray); *n* = 3 with representative image shown, scale bar: 20 μm (columns 1–3) and 10 μm (last column is a higher-resolution image of tissue denoted by yellow box). (**F**) Volcano plot for ether lipids in pancreatic tissue from 30-week-old mice represented as Pex5^Pdx1–/–^/Pex5^CON^; *n* = 9–11. (**G**) Serum triglyceride content in 15-week-old mice; *n* = 8–10. (**H**) ITT in 10–16-week-old mice; *n* = 8. (**I**) *Pex5* mRNA abundance by RT-PCR from Pex5^CON^ and Pex5^Ins1–/–^ mice; *n* = 6–8. (**J**) Pex5 abundance by immunoblot in isolated islets; *n* = 3. (**K**) Pancreatic tissue costained for Acox1 (green), PMP70 (red), insulin (blue), and Hoechst (gray); *n* = 3 with representative image shown, scale bar: 20 μm. (**L**) Serum triglyceride content in 15-week-old mice; *n* = 10–14. (**M**) ITT in 16–20-week-old mice; *n* = 20. (**A**–**M**) Male mice; ns, not significant; **P < 0.01; ****P < 0.0001. *P* values shown on graph represent AUC calculations. One-way ANOVA with multiple comparisons (**A** and **I**) or 2-tailed Student’s *t* test (**G** and **L**).

**Figure 2:**
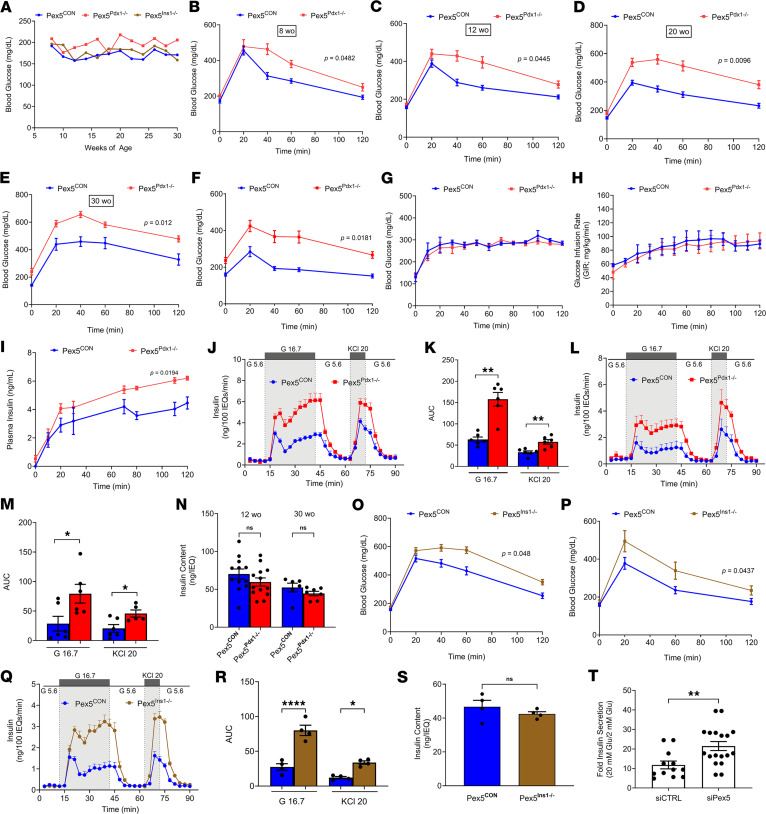
Peroxisomes are critical for maintenance of glucose tolerance and β cell function in male mice. (**A**) Fed blood glucose values in male Pex5^CON^, Pex5^Pdx1–/–^, and Pex5^Ins1–/–^ mice from 8 to 30 weeks of age; *n* = 26–34. (**B**–**E**) i.p. GTTs in 8- (*n* = 9–14), 12- (*n* = 22), 20- (*n* = 15–16), and 30- (*n* = 8–19) week-old mice. (**F**) Oral GTT in 15-week-old mice; *n* = 11–25. (**G**–**I**) Fasting blood glucose, glucose infusion rate, and plasma insulin during a hyperglycemic clamp in 18-week-old mice; *n* = 4. (**J**–**M**) Glucose-mediated (16.7 mM glucose; G 16.7) and KCl-mediated (20 mM; KCl 20) insulin secretion and corresponding AUC calculations in isolated islets from 12- and 30-week-old mice, respectively; *n* = 6. (**N**) Insulin content in islets used for experiments in **J**–**M**; *n* = 6–13. (**O**) i.p. GTT in 30-week-old male Pex5^CON^ and Pex5^Ins1–/–^ mice; *n* = 29–31. (**P**) Oral GTT in 42-week-old mice; *n* = 9. (**Q** and **R**) Glucose-mediated (16.7 mM glucose; G 16.7) and KCl-mediated (20 mM; KCl 20) insulin secretion and corresponding AUC calculation in isolated islets from 30-week-old mice; *n* = 4. (**S**) Insulin content in islets used for the experiment in **Q** and **R**. (**T**) GSIS (2 mM versus 20 mM glucose) in 832/13 cells treated for 48 hours with siRNA control (siCTRL) or siRNA duplexes targeting the Pex5 gene (siPex5) prior to glucose treatment; *n* = 3 individual experiments with 4 replicates for each experiment. ns, not significant; *P < 0.05; **P < 0.01; ****P < 0.0001. *P* values shown on graph represent AUC versus controls. One-way ANOVA with multiple comparisons (**K**, **M**, **N**, and **R**) or 2-tailed Student’s *t* test (**S** and **T**); wo, weeks old.

**Figure 3:**
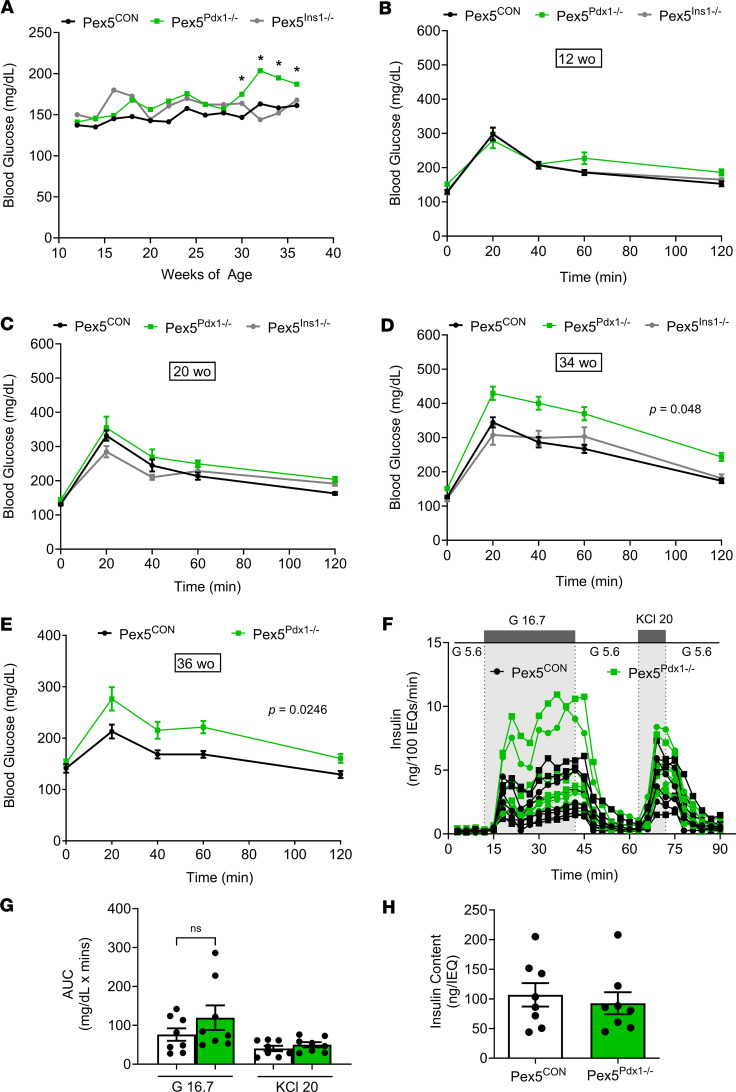
Reduced peroxisomal function in the β cells of female mice has no impact on β cell function. (**A**) Fed blood glucose values in female Pex5^CON^, Pex5^Pdx1–/–^, and Pex5^Ins1–/–^ mice from 12 to 36 weeks of age; *n* = 12–23. (**B**–**D**) Intraperitoneal GTTs in 12-week-old (*n* = 10–20), 20-week-old (*n* = 10–20), and 34-week-old (*n* = 10–20) mice, respectively. (**E**) Oral GTT in 36-week-old mice; *n* = 8–11. (**F** and **G**) Glucose- (16.7 mM glucose; G 16.7) and KCl- (20 mM; KCl 20) mediated insulin secretion, and corresponding AUC calculation, in isolated islets from 36-week-old mice; *n* = 8. (**H**) Insulin content in islets used for experiment in **F** and **G**. ns, not significant; *P < 0.05. *P* values shown on graph represent AUC for Pex5^CON^ versus Pex5^Pdx1–/–^ groups. Multiple unpaired *t* tests (**A**), 1-way ANOVA with multiple comparisons (**G**), or 2-tailed Student’s *t* test (**H**); wo, weeks old.

**Figure 4:**
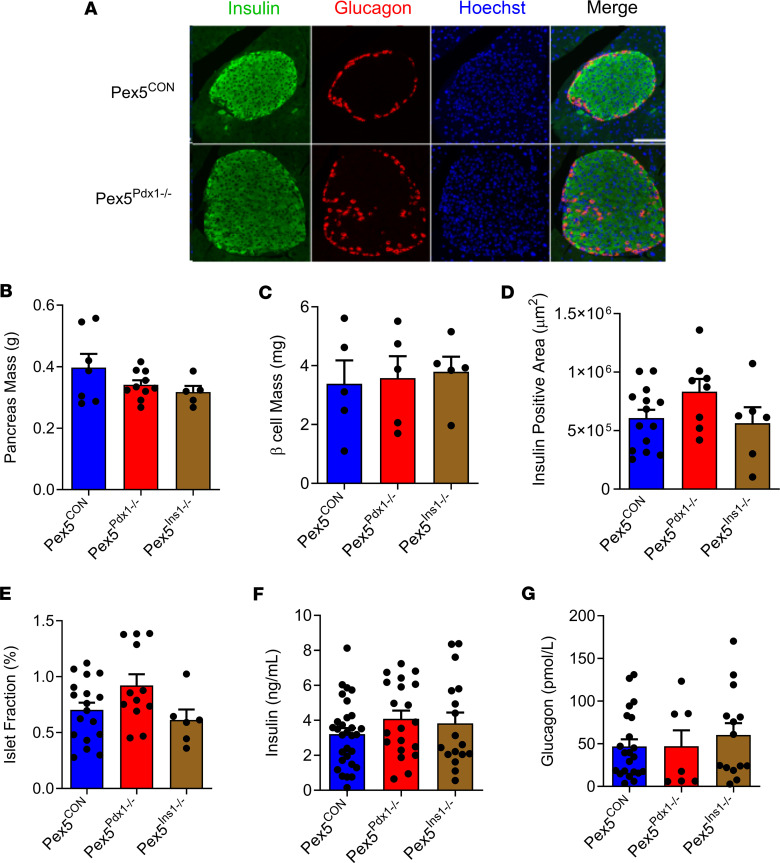
Reduced peroxisomal function in the pancreas of male mice has no impact on islet architecture or hormone content. (**A**) Pancreatic tissue from 30-week-old male Pex5^CON^ and Pex5^Pdx1–/–^ mice costained for insulin (green), glucagon (red), and Hoechst (blue), *n* = 5 with representative image shown, scale bar: 100 μm. (**B** and **C**) Pancreas mass (*n* = 5–10) and β cell mass (*n* = 5), respectively, in 30-week-old mice. (**D** and **E**) Insulin-positive area and islet fraction, respectively, in 30 week-old mice; *n* = 6–14. (**F**) Fasting serum insulin in 30-week-old mice; *n* = 15. (**G**) Fasting serum glucagon in 30-week-old mice; *n* = 7–22. One-way ANOVA (**B**–**G**).

**Figure 5:**
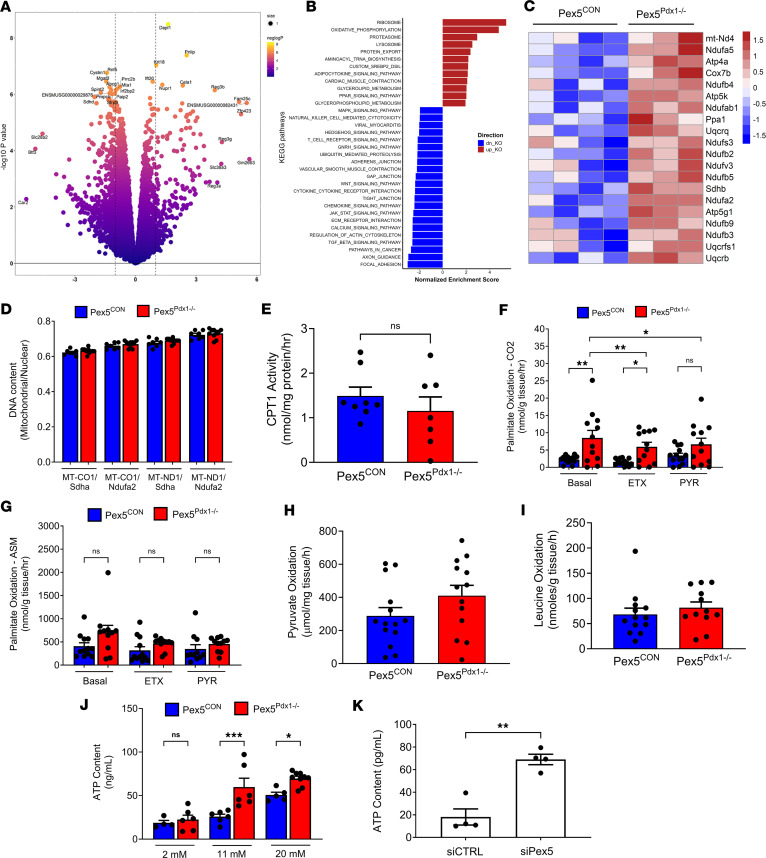
Loss of peroxisomal function correlates with increased oxidative phosphorylation pathway activity, palmitate oxidation, and ATP content in male mice. (**A**–**C**) Volcano plot, KEGG pathway analysis, and upregulated genes in the oxidative phosphorylation pathway, respectively, determined from RNA-Seq data in islets from 22-week-old male Pex5^CON^ and Pex5^Pdx1–/–^ mice; *n* = 3–4. (**D**) Mitochondrial versus nuclear DNA content in isolated islets from 22-week-old mice; *n* = 7–10. (**E**) CPT1 activity in pancreatic mitochondrial isolates from 30-week-old mice; *n* = 7–8. (**F** and **G**) Palmitate oxidation to CO_2_, or acid-soluble metabolites (ASM), respectively, in the basal state, in the presence of etomoxir (ETX) or pyruvate (PYR) in pancreatic homogenates from 30-week-old mice; *n* = 12–13. (**H** and **I**) Pyruvate and leucine oxidation, respectively, in pancreatic homogenates from 30-week-old mice; *n* = 13–14. (**J**) Intracellular ATP content in isolated islets from 30-week-old mice treated with glucose (2, 11, or 20 mM); *n* = 4–9. (**K**) Intracellular ATP in 832/13 cells treated for 48 hours with siCTRL or siPex5 at 11 mM glucose; *n* = 4 individual experiments. ns, not significant; *P < 0.05; **P < 0.01; ***P < 0.001. One-way ANOVA with multiple comparisons (**D**, **F**, **G**, and **J**), or 2-tailed Student’s *t* test (**E**, **H**, **I**, and **K**).

**Figure 6:**
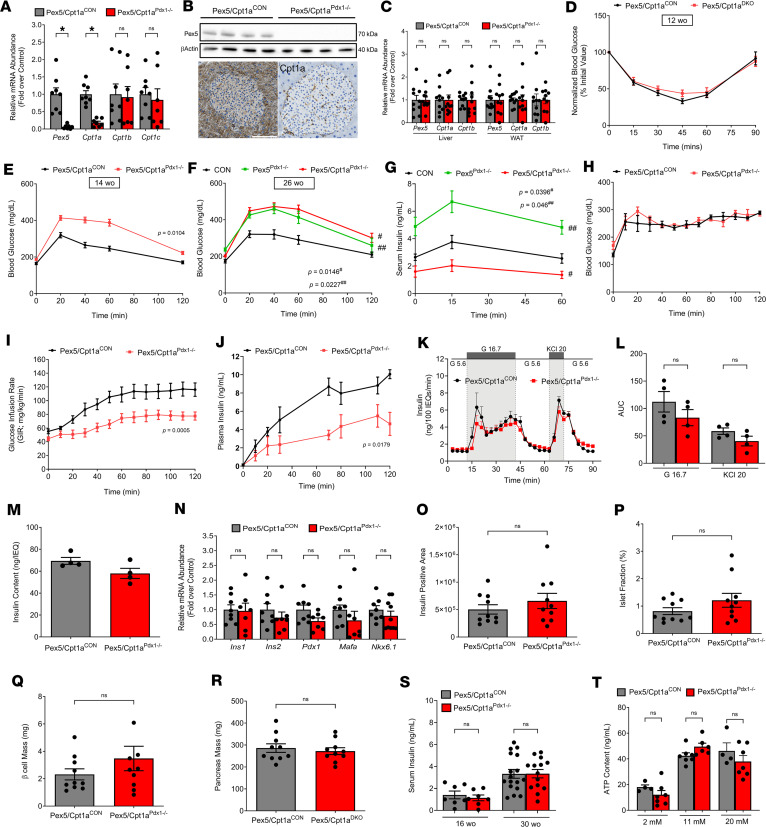
Reduction in CPT1a abundance in peroxisomal-deficient male mice restores β cell GSIS and islet ATP content to control levels. (**A**) mRNA abundance by RT-PCR in islets from male Pex5/Cpt1a^CON^ and Pex5/Cpt1a^Pdx1–/–^ mice; *n* = 7–8. (**B**) Pex5 abundance by immunoblot in isolated islets (top) and CPT1a abundance in pancreatic tissue (bottom), *n* = 4, scale bar: 50 μm. (**C**) mRNA abundance by RT-PCR in liver and scWAT subcutaneous White Adipose Tissue (scWAT); *n* = 10. (**D**) ITT in 12-week-old mice; *n* = 14–18. (**E**) GTT (i.p.) in 14-week-old mice; *n* = 24–36. (**F**) GTT (i.p.) in 26-week-old mice; *n* = 8–16. (**G**) Serum insulin from blood taken at 0-, 15-, and 60-minute time points during GTT shown in **F**; *n* = 12–16. (**H**–**J**) Fasting blood glucose, glucose infusion rate, and plasma insulin, respectively, during hyperglycemic clamp in 18-week-old mice; *n* = 4–5. (**K** and **L**) Glucose-mediated (16.7 mM glucose; G 16.7) and KCl-mediated (20 mM; KCl 20) insulin secretion and corresponding AUC calculation in islets from 15-week-old mice; *n* = 4. (**M**) Insulin content in islets used for the experiment in **K** and **L**. (**N**) mRNA abundance by RT-PCR in islets; *n* = 7–8. (**O**–**R**) Insulin-positive area, islet fraction, β cell mass, and pancreas mass, respectively, in 30-week-old male mice; *n* = 10. (**S**) Serum insulin in 16-week-old (*n* = 7) and 30-week-old (*n* = 14–17) mice. (**T**) ATP content in isolated islets from 30-week-old mice treated with glucose (2, 11, or 20 mM); *n* = 4–8. ns, not significant; *P < 0.05. *P* values shown on graph represent AUC calculations versus controls. One-way ANOVA with multiple comparisons (**A**, **C**, **L**, **N**, **S**, and **T**) or 2-tailed Student’s *t* test (**M** and **O**–**R**).

**Figure 7:**
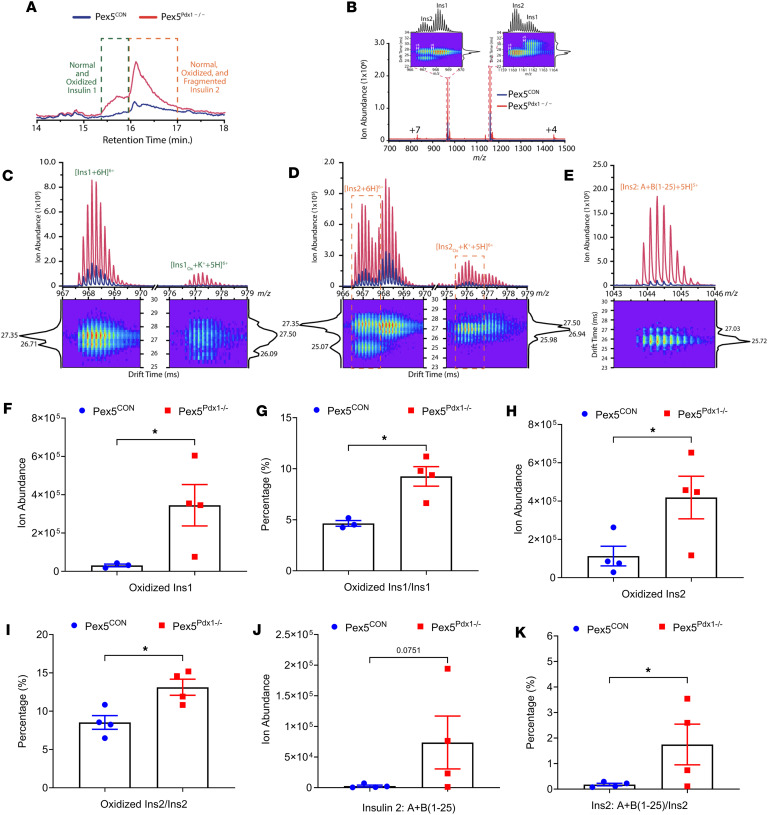
Loss of functional peroxisomes correlates with increased abundance of posttranslationally modified insulin 1 and 2 and a truncated insulin 2 peptide in male Pex5^Pdx1–/–^ mice. (**A**) Liquid chromatography (LC) chromatogram of insulin 1 eluting at 15.4–16 minutes and insulin 2 eluting at 16–17 minutes. (**B**) Entire mass spectrum revealing all insulin 1 and 2 peaks at different charge states and the arrival time distribution of peaks at 966.4 *m/z* and 1,159.5 *m/z*. (**C**–**E**) Mass spectra and arrival time distributions of insulin 1 and oxidized insulin 1; insulin 2 and oxidized insulin 2; and insulin 2: A+B(1–25) fragment, respectively. (**F**) Ion abundance of oxidized insulin 1, (**G**) percentage ion abundance of oxidized insulin 1/insulin 1, (**H**) ion abundance of oxidized insulin 2, (**I**) percentage ion abundance of oxidized insulin 2/insulin 2, (**J**) ion abundance of insulin 2: A+B(1–25) fragment, and (**K**) percentage ion abundance of insulin 2: A+B(1–25) fragment/insulin 2, in islets from 30-week-old Pex5^CON^ and Pex5^Pdx1–/–^ mice. *n* = 4 individual islets per genotype. *P < 0.05. 2-tailed Student’s *t* test (**F**–**K**).

**Figure 8:**
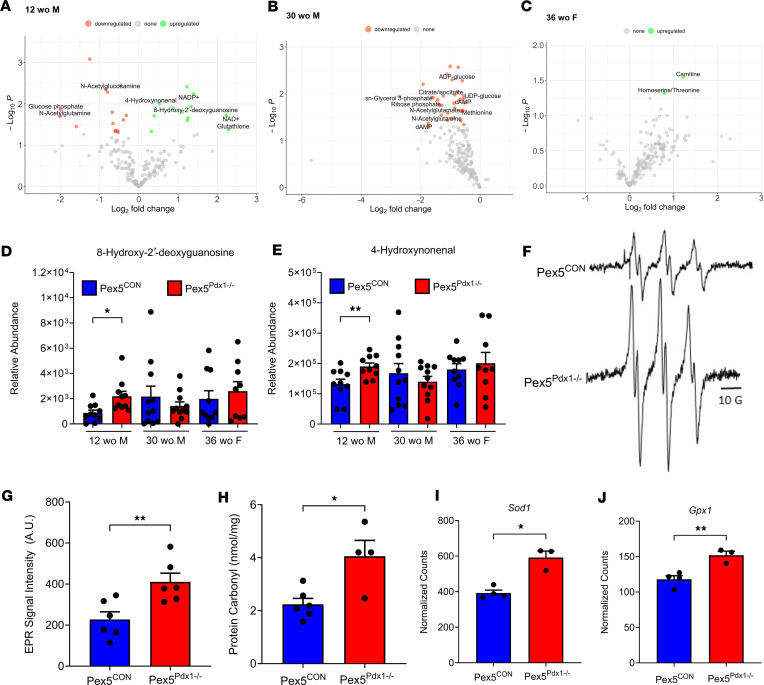
Coordinated alterations in oxidative stress signature in pancreas and islets from Pex5^Pdx1–/–^ mice. (**A**–**C**) Volcano plots for identified metabolites including (**D**) 8-hydroxy-2′-deoxyguanosine, and (**E**) 4-hydroxynonenal in pancreatic tissue from 12-week-old male (**A**), 30-week-old male (**B**), and 36-week-old female (**C**) Pex5^CON^ and Pex5^Pdx1–/–^ mice; *n* = 9–11. (**F**) Representative EPR spectra of carbon-centered lipid-derived radical adducts (POBN-LOO^•^) in pancreatic tissue from 20-week-old male mice and (**G**) bar graphs show EPR signal intensities (arbitrary units) of the measured peak amplitudes normalized to 300 mg tissue. (**H**) Protein carbonylation in islets from 28-week-old male mice. (**I** and **J**) Gene expression, expressed as normalized counts, for the (**I**) *Sod1* and (**J**) *Gpx1* genes in islets from 22-week-old male mice. (**F**–**J**) *n* = 4–6 per genotype. *P < 0.05, **P < 0.01. One-way ANOVA with multiple comparisons (**D** and **E**), or 2-tailed Student’s *t* test (**G**–**J**); wo, weeks old.

**Figure 9:**
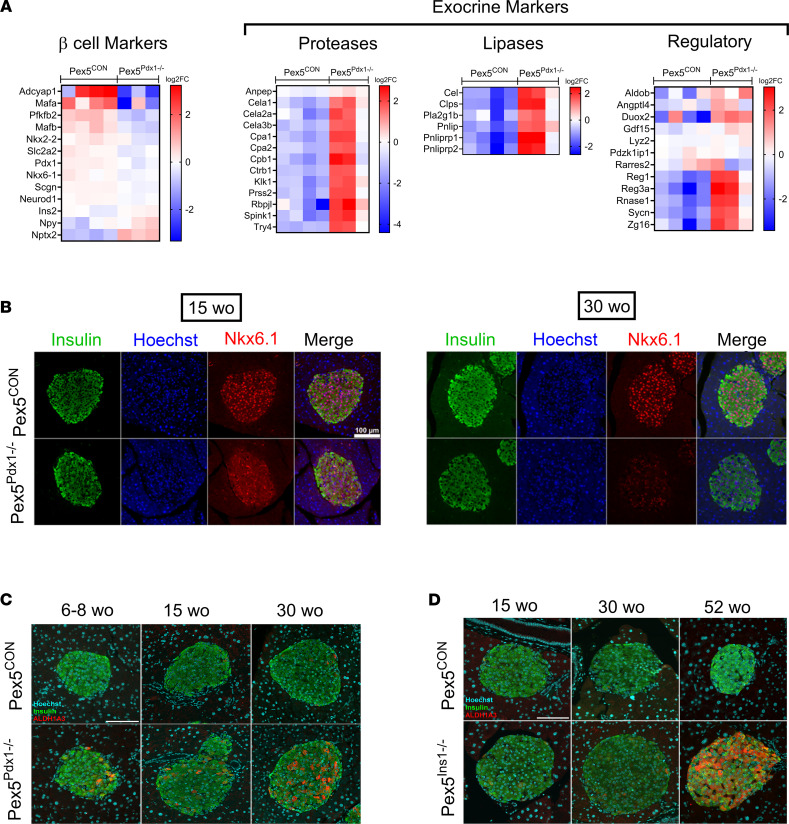
Global shifts in transcriptional signature in islets from male Pex5^Pdx1–/–^ mice. (**A**) Change in expression of β cell and exocrine markers determined from RNA-Seq data in islets from 22-week-old Pex5^CON^ and Pex5^Pdx1–/–^ mice; *n* = 3–4. (**B**) Pancreatic tissue from 15-week-old (left panel) and 30-week-old (right panel) mice costained for insulin (green), Nkx6.1 (red), and Hoechst (blue). (**C**) Pancreatic tissue from 6–8-week-old, 15-week-old, and 30-week-old mice costained for insulin (green), ALDH1A3 (red), and Hoechst (blue). (**D**) Pancreatic tissue from 15-week-old, 30-week-old, and 52-week-old mice costained for insulin (green), ALDH1A3 (red), and Hoechst (blue). (**B**–**D**) *n* = 5 with representative image shown, scale bars: 100 μm; wo, weeks old.

**Figure 10:**
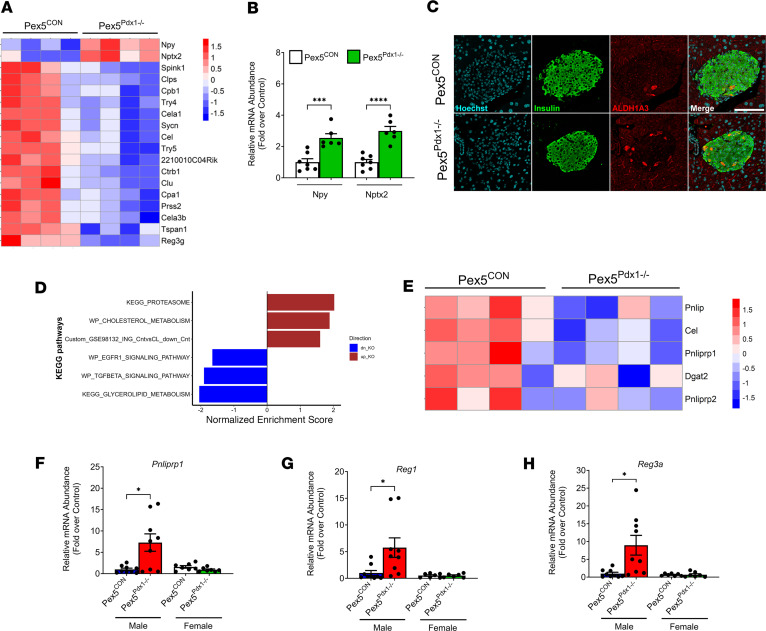
Loss of functional peroxisomes in pancreatic tissue promotes β cell dedifferentiation in female mice. (**A**) RNA-Seq data in islets from 36-week-old female Pex5^CON^ and Pex5^Pdx1–/–^ mice; *n* = 4. (**B**) *Npy* and *Nptx2* mRNA abundance by RT-PCR in islets; *n* = 7. (**C**) Pancreatic tissue from 36-week-old mice costained for insulin (green), ALDH1A3 (red), and Hoechst (blue), *n* = 5 with representative image shown, scale bar: 100 μm. (**D** and **E**) KEGG pathway analysis and expression of genes in the glycerolipid metabolism pathway, respectively, determined from RNA-Seq data in islets from 36-week-old mice; *n* = 4. (**F**–**H**) *Pnliprp1, Reg1,* and *Reg3a* mRNA abundance, respectively, by RT-PCR in islets from male (*n* = 9) and female (*n* = 7) mice. *P < 0.05, ***P < 0.001, ****P < 0.0001. One-way ANOVA with multiple comparisons (**B** and **F**–**H**).

## Version 1 06/30/2026

In-Press Preview

## Version 2 08/10/2026

Electronic publication
