# Supplementary material for: Reduced peroxisomal function increases insulin secretion, promotes insulin oxidation, and impairs β cell maturity
Source: JCI Insight. 2026 Jun 30;11(15):e200202. doi: 10.1172/jci.insight.200202 (PMC13463611; doi:10.1172/jci.insight.200202)
Supplement: Supplementary file 1 [file jciinsight-11-200202-s056.pdf]

Supplementary Figure 1

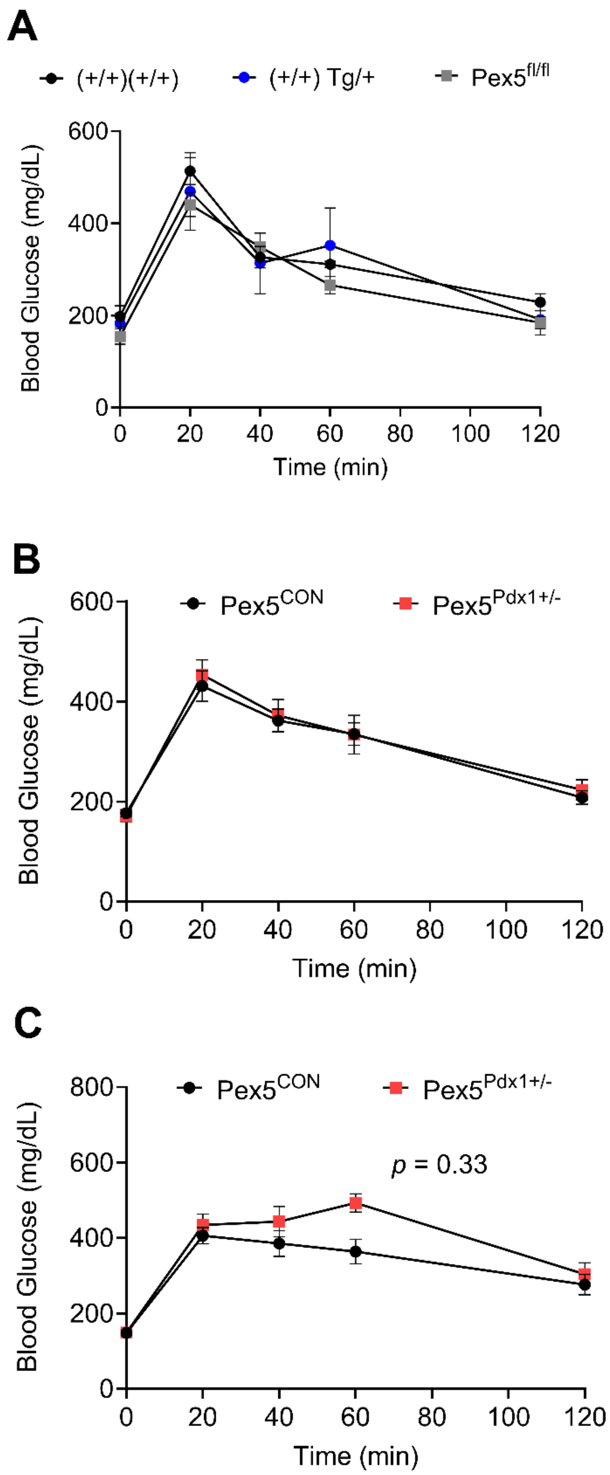

Heterozygous loss of peroxisomal function in the pancreas does not impact glucose tolerance. (A) Intraperitoneal glucose tolerance tests (ipGTTs) and corresponding area under the curve (AUC) calculations in 8 week old male littermate control groups including: C57BL6/J, also referred to here as (+/+)(+/+), Pdx1-cre mice, referred to here as (+/+)Tg/+, and $Pex5^{fl/fl}$ mice; n=5. **(B)** ipGTT in 12 old $Pex5^{CON}$ and heterozygous $Pex5^{Pdx1+/-}$ mice; n=10-20. **(C)** ipGTT in 20 old male $Pex5^{CON}$ and heterozygous $Pex5^{Pdx1+/-}$ mice; n=10-20. Data are shown as mean ± SEM. *p* values shown on graph represent area under the curve (AUC) calculations versus control.

Supplementary Figure 2

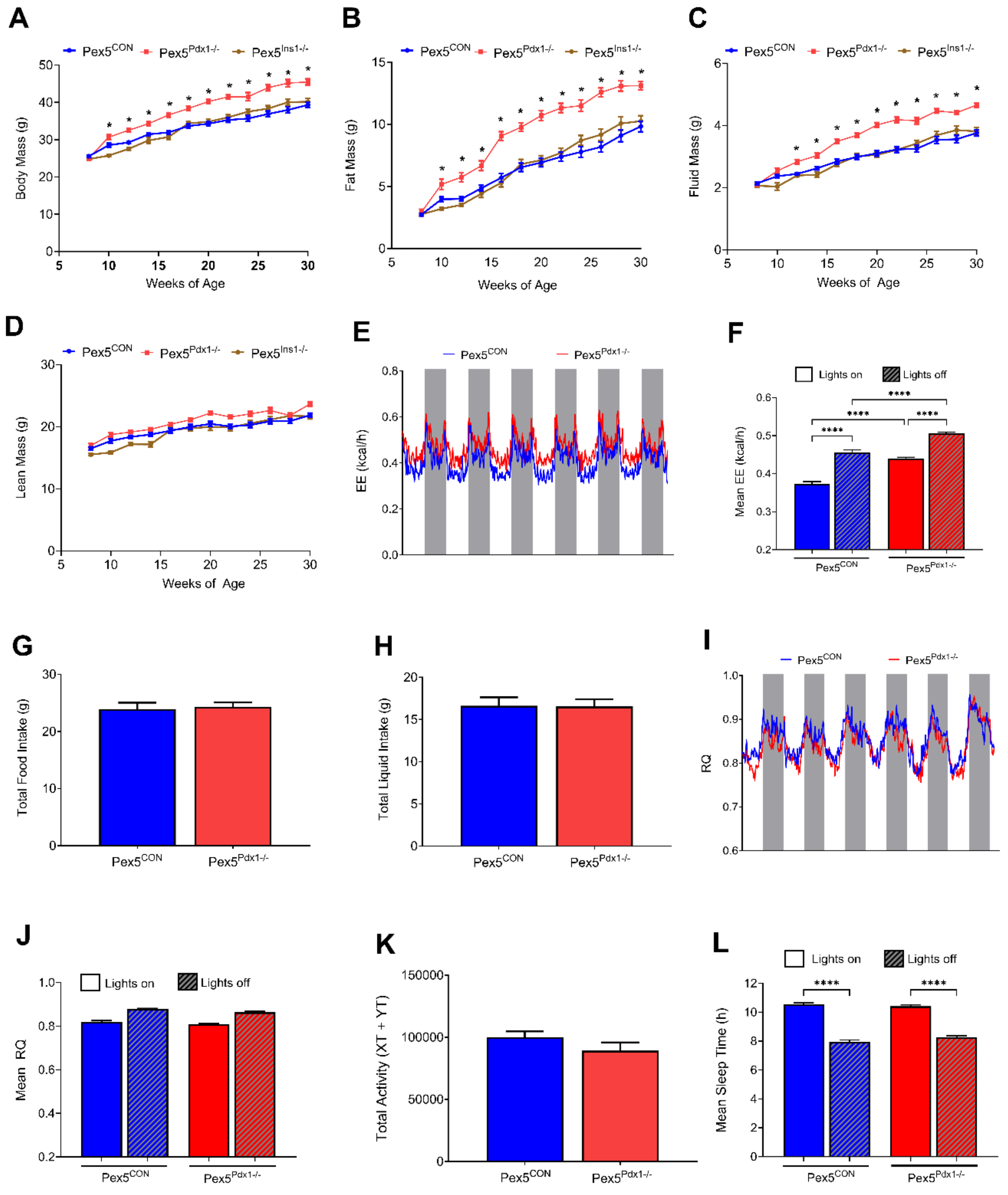

Loss of peroxisomal function in the pancreas of male mice promotes increased body mass, energy expenditure, with no change in respiratory quotient or activity. (A-D) Body mass, fat mass, fluid mass, and lean mass, respectively, in male Pex5^{CON}, Pex5$^{Pdx1-/-}$, and Pex5$^{Ins1-/-}$ mice from 8 to 30 weeks of age; n = 25-30. **(E)** Energy expenditure (EE) across the light (*white bars*) and dark (*gray bars*) cycle over a 7 day period, **(F)** mean EE separated by light on and off cycles for a 7 day period, **(G)** total food intake, **(H)** total liquid intake for a 7 day period, **(I)** respiratory quotient (RQ) across the light (*white bars*) and dark (*gray bars*) cycle over a 7 day period, **(J)** mean RQ calculated by light on and off cycles for a 7 day period, **(K)** total activity measured by X and Y beam breaks, and **(L)** mean sleep time calculated by light on and off cycles for a 7 day period, in 22 week old male Pex5^{CON} and Pex5$^{Pdx1-/-}$ mice; n = 8. Data are shown as mean ± SEM. $^*P < 0.05$, $^{****}P < 0.0001$. Multiple unpaired t tests (**A-D**), one-way ANOVA with multiple comparisons (**F, J, L**), or 2-tailed Student's t test (**G, H, K**).

Supplementary Figure 3

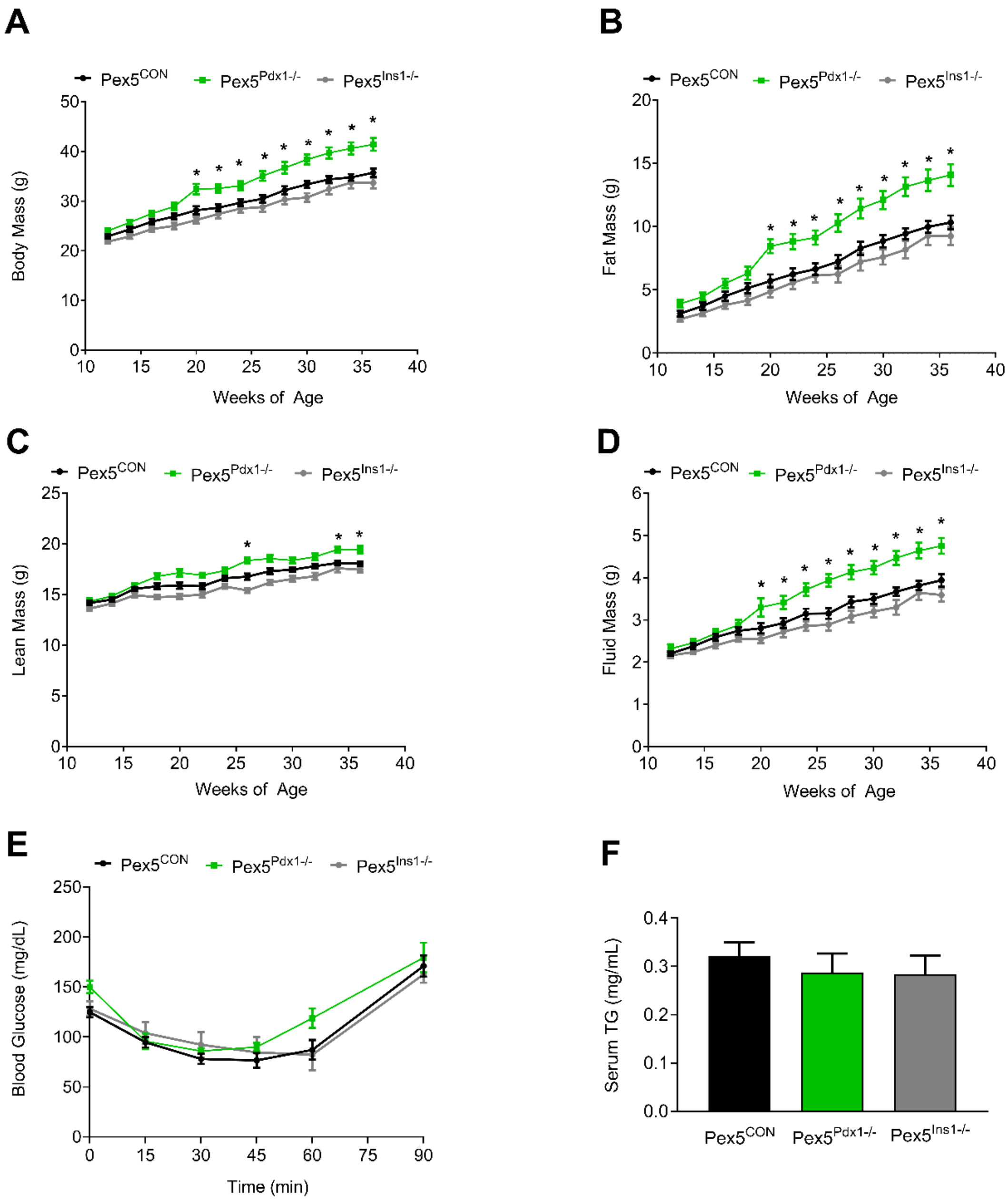

Loss of peroxisomal function in the pancreas and β cells of female mice doesn't alter peripheral insulin sensitivity. (A-D) Body mass, fat mass, lean mass, and fluid mass, respectively, in female Pex5^{CON}, Pex5$^{Pdx1-/-}$, and Pex5$^{Ins1-/-}$ mice from 12 to 36 weeks of age; *n* = 11-19. **(E)** Insulin tolerance test (ITT), and (**F**) serum TGs in 30 week old female Pex5^{CON}, Pex5$^{Pdx1-/-,}$ and Pex5$^{Ins1-/-}$ mice; *n* = 9-20. Data are shown as mean ± SEM. *P < 0.05. Multiple unpaired *t* tests (**A-D**), or one-way ANOVA with multiple comparisons (**F**).

Supplementary Figure 4

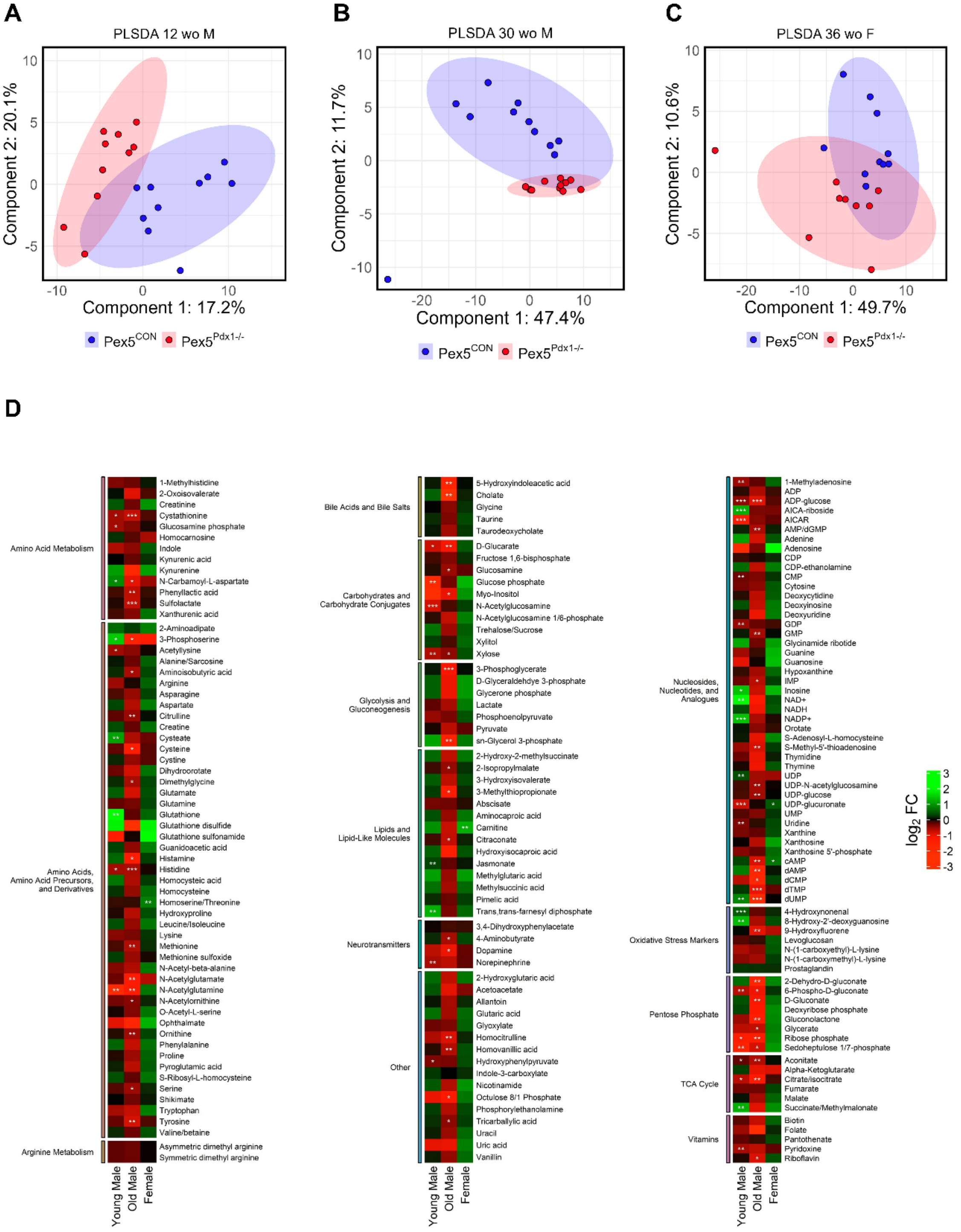

Coordinated alterations in metabolite signature in pancreas from Pex5$^{Pdx1-/-}$ mice. (A-C) PLS-DA scores plot, and (**D**) Heatmap for identified metabolites (data represented as Pex5$^{Pdx1-/-}$/Pex5^{CON}) in pancreatic tissue from 12 wo (young male) and 30 wo (older male) males, and 36 wo female Pex5^{CON} and Pex5$^{Pdx1-/-}$ mice; *n* = 9-11.
