# Supplementary material for: Reduced peroxisomal function increases insulin secretion, promotes insulin oxidation, and impairs β cell maturity
Source: JCI Insight. 2026 Jun 30;11(15):e200202. doi: 10.1172/jci.insight.200202 (PMC13463611; doi:10.1172/jci.insight.200202)

Full unedited gel for Figure 1B

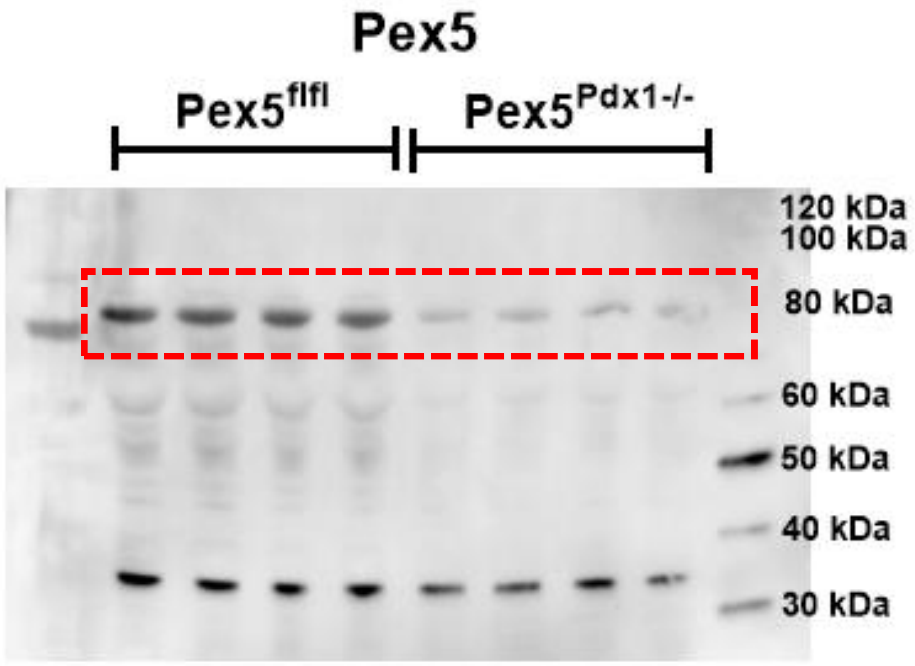

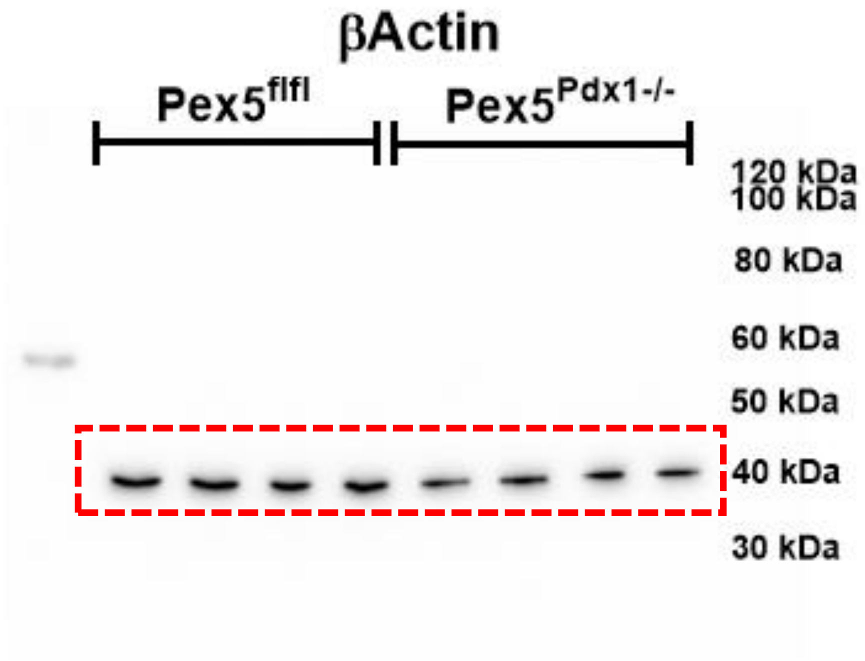

Full unedited gel for Figure 1C

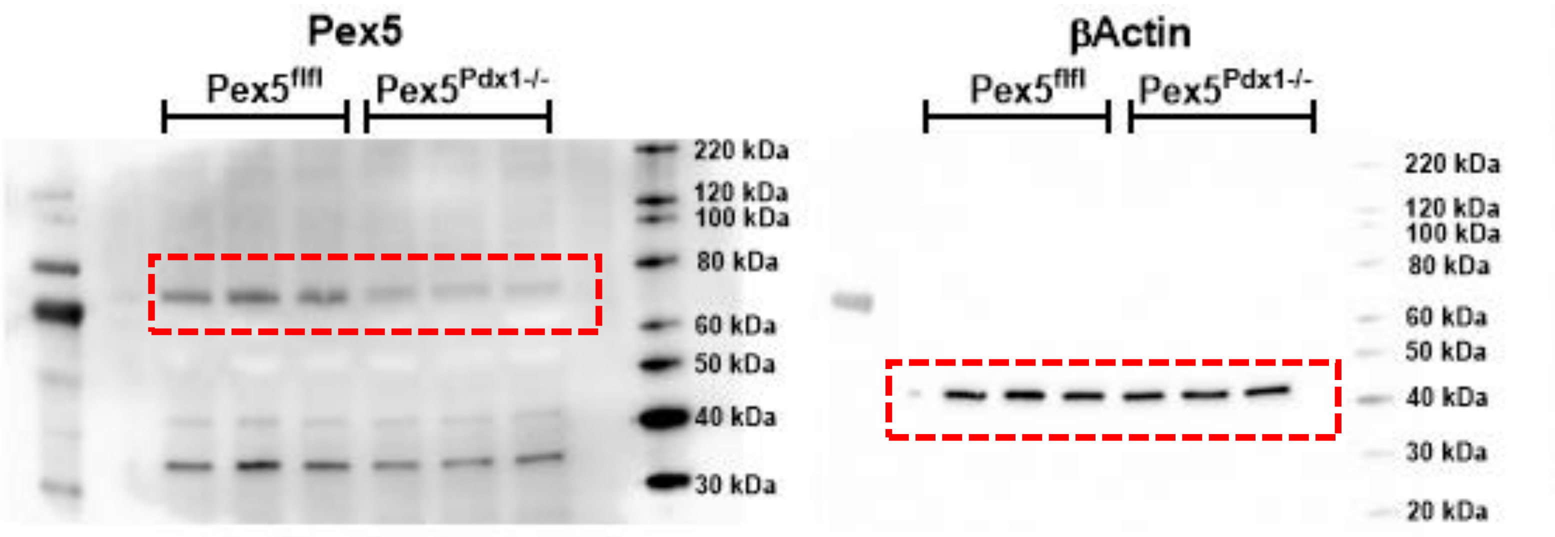

Full unedited gel for Figure 1J

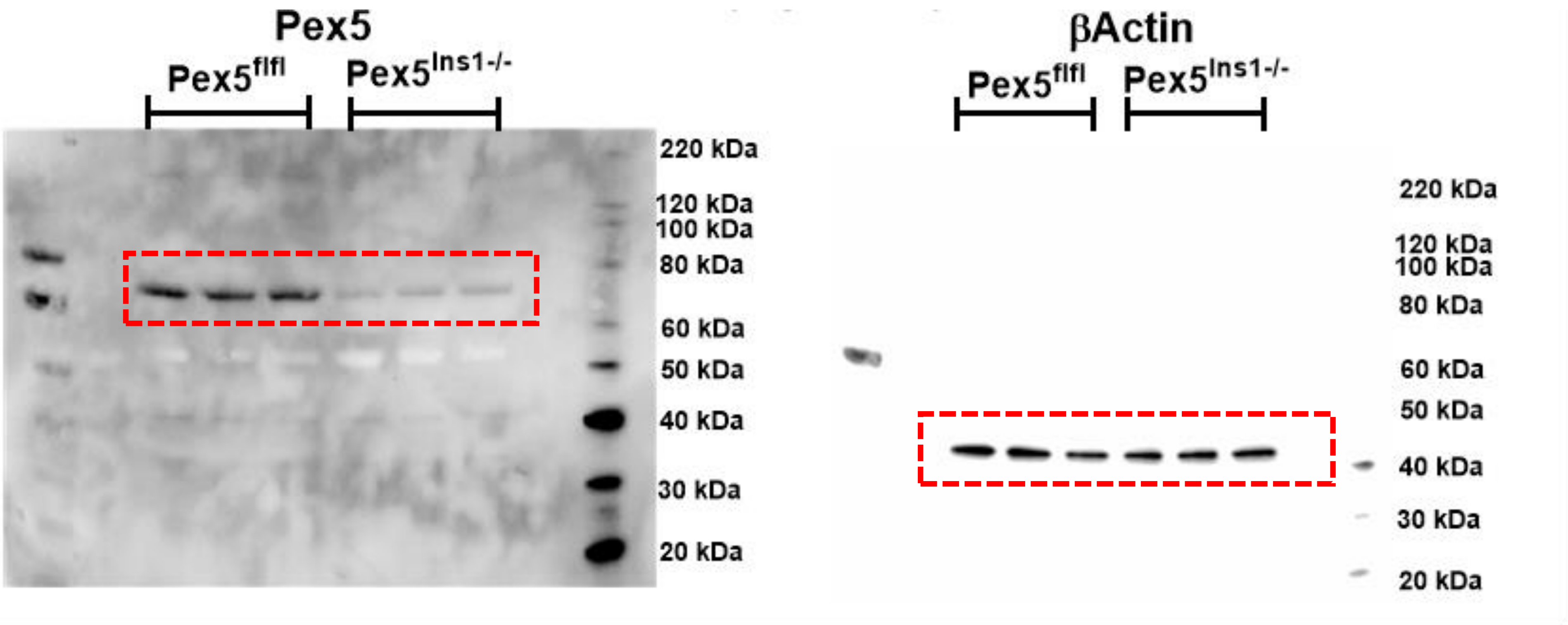

Full unedited gel for Figure 6B

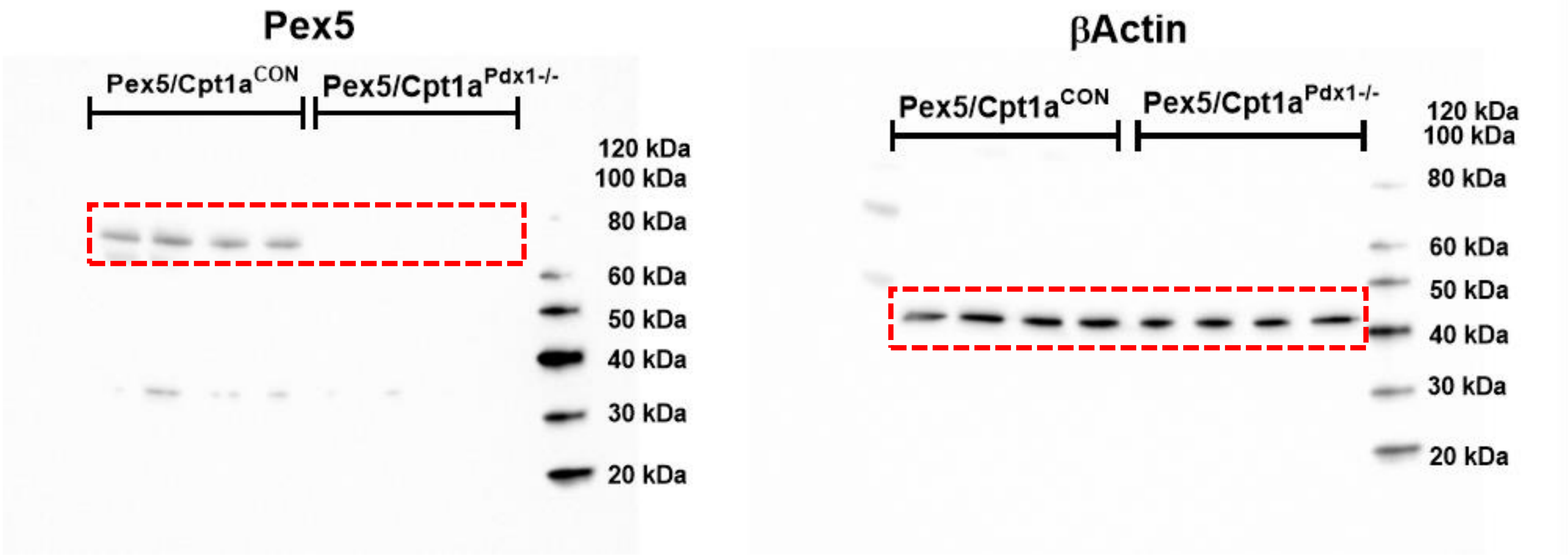

Supplement: Supplementary file 2 [file jciinsight-11-200202-s057.pdf]
